# LncRNA NEAT1 Knockdown Alleviates Macrophage Ferroptosis and Atherosclerosis by Suppressing STAT3 Activation

**DOI:** 10.1155/mi/8862449

**Published:** 2025-11-12

**Authors:** Di Wang, Maomao Zhang, Huiqi Xie, Xiujie Shi, Yang Zheng, Yongxiang Zhang, Yunling Li, Liangqi Chen, Yong Sun, Jian Wu, Bo Yu

**Affiliations:** ^1^Department of Cardiology, The Fourth Affiliated Hospital of Harbin Medical University, Harbin 150001, China; ^2^Key Laboratory of Myocardial Ischemia, Ministry of Education, The Second Affiliated Hospital of Harbin Medical University, Harbin 150086, China; ^3^Department of Cardiology, The Second Affiliated Hospital of Harbin Medical University, Harbin 150086, China

**Keywords:** abundant transcript 1, atherosclerosis, coronary heart disease, ferroptosis, nuclear enriched ubiquitination

## Abstract

**Background:**

This study aimed to investigate the role and mechanism of long noncoding RNA nuclear-enriched abundant transcript 1 (NEAT1) in macrophage ferroptosis during atherosclerosis (AS).

**Methods:**

The clinical characteristics and disease severity were assessed in 84 patients with coronary heart disease (CHD). The role of NEAT1 in high-fat diet-induced AS and the impact of exercise were examined in APOE^−/−^ and NEAT1^−/−^ mice. Human monocyte THP-1 cells were utilized to explore cellular mechanisms underlying AS. Quantitative real-time PCR, immunofluorescence staining, and Western blot analysis were employed to analyze gene expression. Transmission electron microscopy and fluorescence in situ hybridization were used to examine cellular and tissue-level changes. Bioinformatics analyses were conducted to explore protein interactions and functional networks.

**Results:**

NEAT1 expression and iron levels were correlated with disease severity in CHD patients. In THP-1 cells, oxidized low-density lipoprotein (ox-LDL) induced NEAT1 expression, ferroptosis marker ACSL4, reactive oxygen species (ROS), and mitochondrial abnormalities. Knockdown of NEAT1 reversed these effects. NEAT1 overexpression increased pSTAT3, ACSL4, and ROS production, reversed by STAT3 inhibitor. NEAT1 physically interacted with STAT3 via FBXW11. Knockdown of NEAT1 promoted pSTAT3 ubiquitination, reduced ACSL4 expression, and reversed ox-LDL effects. NEAT1 deletion attenuated macrophage ferroptosis and AS in APOE^−/−^ mice. Exercise reduced NEAT1 and ferroptosis indicators in mice and CHD patients.

**Conclusions:**

NEAT1 plays a crucial role in macrophage ferroptosis during AS. Targeting NEAT1 or exercising may provide therapeutic interventions against AS.

## 1. Background

Coronary heart disease (CHD) is the leading cause of death worldwide, with atherosclerosis (AS) representing its pathophysiological hallmark [1, 2]. It is urgent to identify new and effective strategies for AS prevention and intervention [3]. There is increasing evidence that monocytes and macrophages are involved in AS development [4]. Long noncoding (lnc) RNA nuclear-enriched abundant transcript 1 (NEAT1) acts as an immunomodulator in AS patients by affecting monocyte and macrophage functions [5]. NEAT1 facilitates AS progression by promoting the formation of paraspeckles [6]. In addition, NEAT1 knockdown inhibits macrophage lipid accumulation and inflammation to alleviate AS [7]. However, the underlying mechanism remains largely unknown.

A recent study has shown that the iron level in macrophages within AS plaques is significantly higher than in control cells [8]. Iron overload in smooth muscle cells creates a pro-oxidative microenvironment that promotes foam cell development [9]. The lipid peroxidative damage caused by reactive oxygen species (ROS) resulting from iron overload may lead to ferroptosis, an iron-dependent cell death distinct from apoptosis, necrosis, and autophagy [10–12]. Inhibition of ferroptosis can alleviate AS by reducing lipid peroxidation and endothelial dysfunction in aortic endothelial cells [13]. Ferroptosis may, therefore, play an essential role in AS pathogenesis. Intriguingly, inhibition of NEAT1 suppresses ferroptosis in non-small-cell lung cancer and glioma [14, 15]. NEAT1 alleviates ferroptosis in sepsis [16]. Thus, NEAT1 might regulate macrophage ferroptosis and contribute to AS development, but this hypothesis remains untested.

In this study, we examined the role of NEAT1 in macrophage ferroptosis and AS prevention in THP-1 cells and high-fat diet (HFD)-fed APOE^−/−^ mice. Since NEAT1 targets STAT3 and regulates the ubiquitination of STAT family proteins [17–19], we investigated the underlying mechanism in terms of STAT3 activation and ubiquitination. Our study suggests that NEAT1 promotes macrophage ferroptosis through STAT3 activation, serving as a promising therapeutic target in AS.

## 2. Methods

### 2.1. Patients

All participants were of Han ethnicity. A total of 84 patients with CHD were recruited from the Second Affiliated Hospital of Harbin Medical University (Heilongjiang, China) between September 2021 and December 2021. The clinical characteristics of the patients are summarized in Table S1. CHD was defined as ≥50% lumen stenosis of at least one major coronary artery in the most recent coronary angiography [20]. According to international guidelines, all patients received antiplatelet treatment in the hospital [21]. The exercise group consisted of 50 CHD patients who jogged or walked 30–60 min each day, 5 days per week, for more than 3 months after discharge between April 2021 and June 2021. The healthy control group consisted of 50 age-matched participants undergoing physical examinations at the same hospital. The inclusion criteria were as follows: (1) patients with a confirmed diagnosis of CHD based on coronary angiography and (2) for the CHD + exercise group, patients who engaged in consistent home-based exercise following hospital discharge. The exclusion criteria included: (1) history of coronary artery bypass grafting; (2) recent use of medications known to significantly affect lipid metabolism, such as corticosteroids; (3) history of severe renal insufficiency, malignancy, thyroid dysfunction, or autoimmune disease. This study was approved by the Ethics Committee of the Second Affiliated Hospital of Harbin Medical University (Approval Number KY2020-156). Written informed consent was obtained from all participants or their families before participation in the study. Human tissue samples were examined following the Declaration of Helsinki. CHD severity was assessed using the Gensini [22] scoring system (Table 1).

### 2.2. Animals

The animal study was approved by the Institutional Animal Care and Use Committee at the Second Affiliated Hospital of Harbin Medical University (sydwgzr2020-095). All procedures were conducted following the guidelines of Directive 2010/63/EU of the European Parliament on animal protection. Eight-week-old male APOE^−/−^ mice and NEAT1^−/−^ mice weighing approximately 17 g were purchased from Beijing Vital River Laboratory Animal Technology Co. Ltd. (China) and Cyagen Biosciences (Guangzhou, China), respectively. NEAT1^−/−^ mice were crossed with APOE^−/−^ mice and identified as APOE^−/−^NEAT1^−/−^ mice according to the results of genotyping using the primers summarized in Table S2.

To investigate the role of NEAT1 in HFD-induced AS, mice were randomly assigned to three groups: APOE^−/−^ control, APOE^−/−^ + HFD, and APOE^−/−^NEAT1^−/−^ + HFD (*n* = 8/group). To investigate the effect of exercise on HFD-induced AS, mice were randomly assigned to APOE^−/−^ control, APOE^−/−^ + HFD, and APOE^−/−^ + HFD + exercise groups (*n* = 8/group). In a 16-week diet intervention, APOE^−/−^ control mice were fed a normal diet, while the other groups were given an HFD containing 79.25% carbohydrates, 16.75% fat, and 4% protein. Each mouse consumed 4–8 g of food per day. The APOE^−/−^ + HFD + exercise group performed aerobic exercise on a treadmill once a day, 5 days a week. The exercise consisted of a 10 min warm-up at 10 m/s, a 40 min exercise at 16 m/s, and a 10 min recovery at 10 m/s.

### 2.3. Animal Sample Collection

Blood samples and AS plaques were collected from the mice at 16 weeks after the diet/exercise intervention. Blood samples were obtained via retro-orbital vein puncture and then transferred to an anticoagulation tube. To anesthetize the mice, 0.806 g tribromoethyl alcohol (2,2,2-Tribromoethanol; HY-B1372, MedChemExpress, NJ, USA) was dissolved in 500 U tert-amyl alcohol overnight, mixed with 39.5 mL of saline on the next day, and injected intraperitoneally at 0.1 mL per 10 g body weight [23, 24]. For euthanasia, tribromoethyl alcohol was used. The heart and aorta were immediately harvested. The heart was embedded in OCT and sectioned using a freezing microtome. The tissue sections were stored at −40°C until use. Aortic plaques were obtained and frozen in liquid nitrogen.

### 2.4. Cell Culture and Treatment

THP-1 human monocytes were purchased from Procell Life Science and Technology (Wuhan, Hubei, China) and cultured in RPMI-1640 medium containing 10% fetal bovine serum (Excell FSP500, USA) in a humidified atmosphere with 5% CO_2_ at 37°C. Before drug treatment or transfection, THP-1 cells were exposed to 50 ng/mL phorbol-12-myristate-13 acetate (PMA; Sigma-Aldrich, St. Louis, MO, USA) for 48 h for polarization [25]. THP-1 cells were treated with 5 µM erastin (HY-15763; MedChemExpress, NJ, USA), 5 µM RSL3 (HY-100218A; MedChemExpress), 2 µM iron (450278; Merck-Sigma), or 1 µg/mL LPS (L2630; Merck-Sigma). To optimize oxidized low-density lipoprotein (ox-LDL) treatment, cells were treated with different concentrations of ox-LDL (50, 100, or 200 µg/mL, YB002; Yiyuan Biotechnology, China) for 24 h or with 50 µg/mL of ox-LDL for different durations (8, 24, or 48 h). For ferroptosis inhibition, cells were exposed to 10 µM ferrostatin-1 (fer-1, HY-100579; MedChemExpress) for 24 h. For ubiquitination investigation, cells were treated with 10 µM proteasome inhibitor MG-132 (HY-13259; MedChemExpress) for 6 h. To optimize STAT3 inhibition, cells were exposed to different concentrations of STAT3 inhibitor stattic (1, 2, 4, 8, or 20 µM, HY-13818; MedChemExpress) for 24 h or to 1 µM stattic for different durations (2, 4, 8, 12, or 24 h).

The RAW264.7 macrophage mouse cell line was used for immunoprecipitation and gene silencing experiments to study the role of NEAT1 in regulating ox-LDL-induced ferroptosis and STAT3 ubiquitination in AS. Cells were obtained from ATCC/ScienCell/the Shanghai Cell Bank (YS3639C; Shanghai, China) and cultured in high-glucose DMEM medium supplemented with 10% fetal bovine serum and 1% penicillin-streptomycin in a humidified atmosphere of 5% CO_2_ at 37°C.

### 2.5. Cell Transfection

THP-1 cells were plated in 6-well plates at 3 × 10^5^ cells/well and cultured in serum-free medium for 24 h. Cells were transfected with 10 µL small interfering RNA (siRNA; 100 nM) against NEAT1 or 5 µg NEAT1-overexpressing vectors using Lipofectamine 3000 (L3000015; Thermo Fisher Scientific, Waltham, MA, USA) following the manufacturer's protocol. Scramble siRNA and empty vectors were used as negative controls. The medium was replaced at 8 h after transfection, and cells were cultured for 24 h before treatment.

### 2.6. Isolation of Peripheral Blood Mononuclear Cells (PBMCs)

PBMCs were isolated from human and mouse peripheral blood samples using PBMC separation kits (TBD2011H, TBD2011M; Tianjin Haoyang Biological Manufacture, China) following the manufacturer's protocols. Briefly, the diluted blood samples were mixed with monocyte extraction solution and centrifuged at 2500 rpm for 25 min. The monocyte fraction was transferred to a new tube and centrifuged for 5 min at 1000 rpm. Cells were resuspended in PBS solution, followed by centrifugation at 1000 rpm for 5 min. The PBMCs were stored at −80°C until use.

### 2.7. Hematoxylin and Eosin (HE) Staining

The mouse aortic AS plaque samples were fixed with 4% paraformaldehyde for 10 min and washed 3 times with water for 5 min each. After staining with hematoxylin for 5 min, the nuclei were washed with water for 10 min. Afterward, the section was stained with eosin for 3 min, followed by 10–20 s of washing with water. The sections were then sequentially treated with alcohol (75%, 95%, and 100%, each for 1 min) and xylene (I, II, and III, each for 2 min). The sections were mounted with neutral balsam.

### 2.8. Oil Red O Staining

The mouse aortic AS plaque sections were rinsed with 60% isopropanol for 10 s before staining with Oil Red O for 30 min. The nuclei were stained with hematoxylin for 5 min.

### 2.9. Masson Staining

The mouse aortic AS sections were incubated with mordant dye for 1 h at 56°C and washed with water for 10 min afterward. The sections were sequentially stained with celestine blue (2 min, twice), Mayer hematoxylin (2 min, twice), acidic ethanol (until the tissue became red), Li Chunhong acid fuchsin (10 min, twice), phosphomolybdic acid (10 min), and aniline blue (5 min). After each staining, the sections were washed with water for 10 s. Excess aniline blue solution was removed with weak acid for 2 min. The sections were then sequentially treated with alcohol (75%, 95%, and 100%, each for 1 min) and xylene (I, II, and III, each for 2 min) and mounted using neutral balsam.

### 2.10. Modified Prussian Blue Staining

After staining the mouse aortic AS plaque sections with Prussian blue for 30 min, the sections were washed with water for 1 min. The sections were blocked with 99.7% methanol for 20 min and washed with water for 5 min. The procedure was repeated three times. The sections were stained with DAB for 15 min and nuclear fast red for 8 min. After sequential treatments with alcohol (75%, 95%, and 100%, each for 1 min) and xylene (I, II, and III, each for 2 min), the sections were mounted with neutral balsam. Iron appeared reddish brown [26].

### 2.11. Quantitative Real-Time PCR (qRT-PCR)

Total RNA was isolated using Trizol (15596-026; Invitrogen, USA) following the manufacturer's instructions. cDNA was synthesized using a Roche reverse transcription kit according to the manufacturer's protocol. PCR was performed on a Corbett instrument. The sequences of the primers are summarized in Table S2. Relative gene expression was calculated using the 2^−*ΔΔ*CT^ method.

### 2.12. Measurement of ROS

Intracellular and plasma ROS levels were measured using a ROS assay kit (S0033S, Beyotime, Shanghai, China) containing dichlorodihydrofluorescein diacetate (DCFH-DA). Briefly, plasma and cell samples were incubated with DCFH-DA for 30 min at 37°C. The fluorescence intensities at 488 mm and 525 mm were measured using a microplate reader (Tecan, Switzerland). Tissue ROS level was measured using a BBOxiProbe kit (BB-470512; BestBio, Shanghai, China) containing DCFH-DA following the manufacturer's instructions. Briefly, the tissue sample was incubated with DCFH-DA for 60 min at 37 °C in the dark. Images were acquired using confocal laser microscopy (Leica Microsystems, Wetzlar, Germany).

### 2.13. Measurement of Malondialdehyde (MDA), Lipid Peroxidation, and Iron

MDA was measured using a lipid peroxidation MDA assay kit (S0131S; Beyotime) following the manufacturer's instructions. The optical density was measured with a microplate reader at 532 nm. Lipid peroxidation was measured using L248 Liperfluo staining (Dojindo, Beijing). Cells were incubated with 5 µM Liperfluo for 30 min at 37°C. The plasma iron level was measured using an iron colorimetric assay kit (E1042; Pulilai, Beijing) following the manufacturer's instructions. The optical density was determined at 550 nm using a microplate reader. Intracellular iron staining was performed using FerroOrange (Dojindo, Beijing) following the manufacturer's instructions. Images were captured using confocal laser microscopy.

### 2.14. Flow Cytometry

Flow cytometry was used to determine intracellular lipid peroxidation. The cell suspension was centrifuged, resuspended in PBS, and then centrifuged again at 1000 rpm for 5 min. Cells were incubated with 2 µM BODIPY-C11 (D3861; Thermo Fisher Scientific). The fluorescence intensity was measured using a FACSAria III cell sorter (BD, Franklin Lakes, NJ, USA).

### 2.15. Immunofluorescence Staining

Aortic plaque tissue sections were incubated with 0.5% Triton for 20 min. After blocking with 5% bovine serum albumin for 30 min, the sections were incubated with primary antibodies against actin (TA-09; Zhongshan Golden Bridge, China), acyl-CoA synthetase long-chain family member 4 (ACSL4; #155282; Abcam, Cambridge, UK), GPX4 (AF-DF6701, Affinity, China), FBXW11 (k005461p; Solarbio, Beijing, China), STAT3 (9139S; Cell Signaling Technology, Danvers, MA, USA), pSTAT3 (Y705) (ab76315; Abcam), ubiquitin (ab179434; Abcam), and CD68 (FA-11) (ab53444; Abcam) for 8 h at 4°C. The sections were then incubated with goat anti-rabbit IgG H&L (Alexa Fluor 647, ab150083; Abcam), goat anti-mouse IgG H&L (Alexa Fluor 488, ab150113; Abcam), goat anti-rat IgG H&L (Alexa Fluor 488, ab150157; Abcam), or goat anti-mouse IgG H&L (Alexa Fluor 647, ab150115; Abcam) for 1 h at room temperature. The nuclei were stained with DAPI. Images were acquired using confocal laser microscopy.

### 2.16. RNA Binding Protein Immunoprecipitation Assay (RIP)

RIP was performed using a RIP assay kit (KT102-01; Guangzhou Saicheng, China) following the manufacturer's protocol. Briefly, cells were collected and lysed using RIP lysis buffer. The cell lysates were incubated with RIP buffer containing magnetic beads conjugated to anti-STAT3 antibody (9139S; Cell Signaling Technology). The samples were incubated with proteinase K to digest proteins, and then the immunoprecipitated RNA was isolated. NEAT1 was detected by qRT-PCR in purified RNA. The total RNAs were the input controls.

### 2.17. Co-Immunoprecipitation (Co-IP)

Total proteins were isolated from THP-1 cells. The agarose beads (B23202; Bimake, Canada) were incubated with the primary antibody for 4 h at 4°C with continuous mixing. Then, the protein samples (1 mg) were incubated with the beads/antibody mixture on a shaker for 2–4 h at 4°C. Equal amounts of proteins were separated on a 10% SDS-PAGE gel and transferred onto polyvinylidene fluoride membranes. After blocking with 5% skim milk in Tris-buffered saline for 1 h at room temperature, the membranes were incubated with primary antibodies against actin (TA-09; Zhongshan Golden Bridge, China), ACSL4 (#155282; Abcam, Cambridge, UK), FBXW11 (k005461p; Solarbio, Beijing, China), STAT3 (9139S; Cell Signaling Technology, Danvers, MA, USA), pSTAT3 (Y705) (ab76315; Abcam), and ubiquitin (ab179434; Abcam) at 4°C overnight. The membranes were then incubated with secondary antibodies (ZB-2305, ZB-2301; SGB-Bio, China; 1:10,000) for 1 h at room temperature. Images were acquired using ImageNote.

### 2.18. Western Blot Analysis

THP-1 cells were lysed in RIPA buffer (P0013B; Beyotime). After centrifuging at 4°C and 12,000 r/min for 15 min, the supernatant was collected. Protein concentration was measured using the BCA assay (P0012; Beyotime). A total of 20 µg proteins were separated on a 10% SDS-PAGE gel and then transferred to a polyvinylidene fluoride membrane. After blocking with 5% skim milk for 1 h at room temperature, the membrane was incubated with the primary antibodies mentioned in the immunofluorescence staining method. After washing with Tris-buffered saline containing 0.1% Tween 20, the membrane was incubated with a secondary antibody (ZB-2305, ZB-2301; SGB-Bio, China) for 1 h at room temperature. The protein bands were visualized using a Tanon 5100 imaging system (Tanon Science and Technology, Shanghai, China) and analyzed using ImageNote.

### 2.19. Fluorescence In Situ Hybridization (FISH)

The FISH assay was conducted to detect NEAT1 expression in mouse aortic plaques using a FISH kit (GenePharma, Suzhou, Jiangsu, China) following the manufacturer's instructions. In brief, the tissue sections were fixed and dehydrated, followed by incubation with NEAT1 probe (Genepharma) for 12 h at 37°C. Images were acquired using a fluorescence microscope (Leica Microsystems).

### 2.20. Cell Counting Kit-8 (CCK-8) Assay

CCK-8 assay was performed to determine cell viability. THP-1 cells were seeded in 96-well plates and cultured overnight, followed by the corresponding treatment. Cells were incubated with 10 μL CCK-8 solution (HY-K0301; MedChemExpress) for 1–4 h at 37°C. The absorbance was measured at 450 nm using a microplate reader (Tecan).

### 2.21. Transmission Electron Microscope (TEM)

TEM was used to observe the mitochondria of THP-1 cells. Briefly, cells were fixed with immunoelectron microscopy fixative for 5 min and harvested using a cell scraper. After centrifuging at 1000 rpm for 2 min, the fixative was removed, and cells were fixed with fresh fixative for 30 min in the dark at room temperature. Cells were observed, and images were acquired using a Hitachi HC-1 TEM (Hitachi, Japan).

### 2.22. Bioinformatics Analysis

Protein sequences of STAT3, FBXW11, and ACSL4 were obtained from NCBI (https://www.ncbi.nlm.nih.gov/). The 3D structure of each protein was constructed on https://swissmodel.expasy.org/interactive. The structure with the highest recognition degree was selected and optimized using Discovery Studio software. The best model was identified according to the dope score value. The models were assessed using PROCHECK (https://saves.mbi.ucla.edu/). Protein model energy was calculated using ProSA (https://prosa.services.came.sbg.ac.at/prosa.php). Rigid docking was performed within the scope of the natively folded protein. The docking model was established on http://vakser.compbio.ku.edu/resources/gramm/grammx. The protein rigid docking results were presented via the European Protein Biostructure website (https://www.ebi.ac.uk/msd-srv/prot_int/pistart.html). The rigid docking with the lowest binding energy was selected. The potential STAT3-binding proteins that belong to the RING/SCF family were obtained from the ubiquitination annotation databases iUUCD and Chipbase. A protein–protein interaction (PPI) network was constructed to identify the hub proteins. The proteins were scored using Cytoscape software.

### 2.23. Statistical Analysis

Statistical analysis was performed using Prism 7.0 (GraphPad, San Diego, CA, USA) and SPSS Statistics22 (IBM, Armonk, New York, USA). A univariate analysis was used to compare the clinical data among groups. Age was presented as median (interquartile range) and analyzed using the Kruskal–Wallis rank sum test. Lipid levels were presented as mean ± standard deviation and analyzed using statistical tests appropriate to the data distribution. Sex was presented as *n* (%) and analyzed using the chi-square test. Wilcoxon rank sum and chi-square tests with Bonferroni correction were used for pairwise comparisons between multiple groups. Spearman correlation analysis was conducted. *p* < 0.05 was considered statistically significant.

### 2.24. Study Approval

The clinical study has been approved by the Ethics Committee of the Second Affiliated Hospital of Harbin Medical University, China (KY2020-156). All animal studies were approved by the Institutional Animal Care and Use Committee at the Second Affiliated Hospital of Harbin Medical University (sydwgzr2020-095).

## 3. Results

### 3.1. PBMC NEAT1 and Plasma Iron Levels are Associated With the Severity of AS

**Figure 1:**
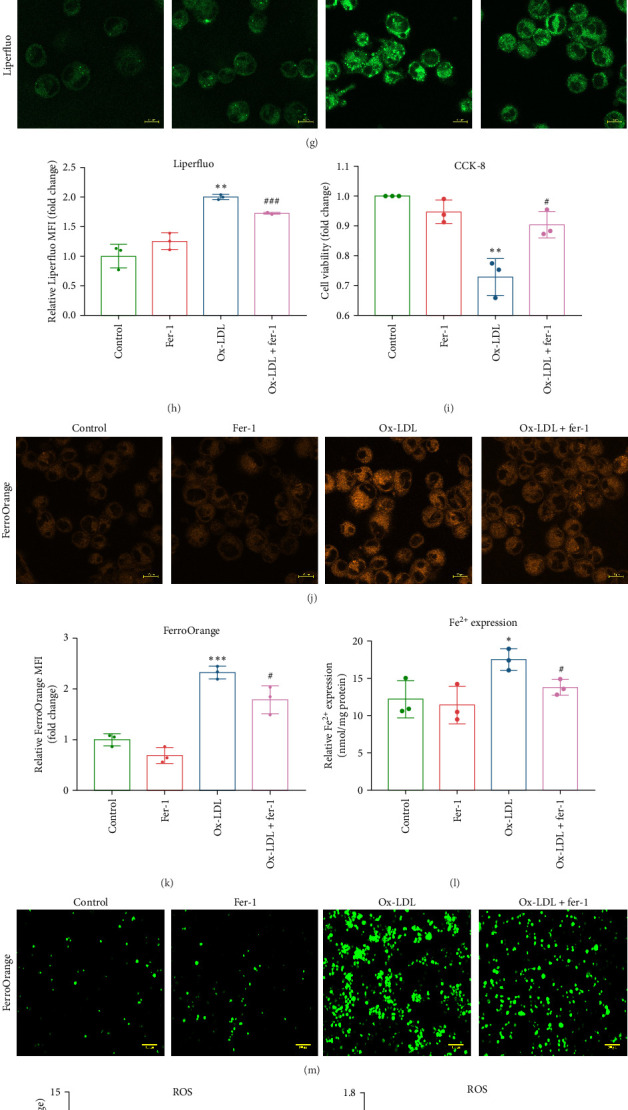
Oxidized low-density lipoprotein (ox-LDL) upregulated NEAT1 expression and induced ferroptosis in THP-1 macrophages. (A) Correlation between PBMCs NEAT1 level and Gensini score of patients. (B) Correlation between plasma iron level and Gensini score. (C) THP-1 cells were stimulated with PMA (50 ng/mL, 48 h) to induce macrophage differentiation, followed by treatment with oxidized low-density lipoprotein (ox-LDL; 100 µg/mL, 24 h) and fer-1 (10 µM, 24 h), alone or in combination. Quantitative real-time PCR (qRT-PCR) was performed to determine the mRNA expression of NEAT1 (*n* = 4). (D) Western blot analysis was conducted to determine the protein expression of ACSL4 and GPX4 (*n* = 3). (E,F) Quantitative analysis of ACSL4 and GPX4 protein. (G) Intracellular lipid peroxidation was detected by LiperFluo. (H) Quantitative analysis of LiperFluo. Scale bar = 10 µm. (I) Cell counting kit-8 (CCK-8) assay. (J) Intracellular iron was detected by FerroOrange. (K) Quantitative analysis of FerroOrange. Scale bar = 10 µm. (L) An iron colorimetric assay kit was used to measure intracellular iron content at 550 nm using a microplate reader. (M) Intracellular reactive oxygen species (ROS) were detected by DCFH-DA. (N) Quantitative analysis of DCFH-DA. Scale bar = 100 µm. (O) A ROS assay kit was used to measure intracellular ROS levels at 488 mm and 525 mm using a microplate reader. (P) Transmission electron microscope (TEM) was used to visualize the mitochondria of THP1 cells. Scale bar = 0.5 µm. (Q) Quantitative analysis of TEM. (R) Flow cytometry analysis of lipid peroxidation reporter C11-BODIPY. (S) Quantitative analysis of C11-BODIPY. Data are expressed as the mean ± standard deviation (SD). *⁣*^*∗*^*p* < 0.05, *⁣*^*∗∗*^*p* < 0.01, *⁣*^*∗∗∗*^*p* < 0.001, *⁣*^*∗∗∗∗*^*p* < 0.0001, vs. control; #*p* < 0.05, ##*p* < 0.01, vs. ox-LDL. MFI, mean fluorescence intensity; ns, nonsignificant.

To explore the involvement of NEAT1 in ferroptosis in AS, we analyzed the association of PBMC NEAT1 and plasma iron levels with the severity of AS. We found that PBMC NEAT1 and plasma iron levels were significantly and positively correlated with the Gensini scores of CHD patients (NEAT1: *r* = 0.4537, *p* < 0.0001; iron: *r* = 0.4264, *p* < 0.0001; Figure 1A,B), suggesting that NEAT1 upregulation and iron overload are associated with AS.

### 3.2. Ox-LDL Induces Ferroptosis and Increases NEAT1 Levels in Macrophages

Iron overload induces ferroptosis [27]. We found that PBMC NEAT1 and plasma iron levels were significantly and positively correlated in CHD patients (*r* = 0.5272, *p* < 0.0001; Figure S1A); thus, we sought to investigate whether NEAT1 is involved in ferroptosis and AS development. We stimulated THP-1 macrophages with ferroptosis inducers (erastin, RSL3, and iron) and AS inducers (ox-LDL and LPS). The results of qRT-PCR showed that all inducers significantly upregulated NEAT1 expression in THP-1 cells compared with control, with ox-LDL exhibiting the strongest effect (Figure S1B). After optimizing the dose and duration of ox-LDL treatment, we treated THP-1 cells with 100 µg/mL ox-LDL for 24 h to achieve maximum NEAT1 upregulation (Figure S1C,D), which could not be reversed by ferroptosis inhibitor fer-1 (Figure 1C).

These data suggest that the upregulation of NEAT1 by ox-LDL in THP-1 cells is a ferroptosis-independent mechanism.

To investigate whether ox-LDL induces ferroptosis, we determined the expression of ferroptosis biomarkers ACSL4 and GPX4 [28, 29] in THP-1 cells exposed to ox-LDL. The results of qRT-PCR (Figure S1E,F) and Western blot analysis (Figure 1D) showed that ox-LDL significantly enhanced ACSL4 expression while attenuating GPX4 expression of THP-1 cells. Fer-1 reversed the effects of ox-LDL on GPX4 expression but not on ACSL4 (Figure 1E,F). Consistently, Liperfluo and FerroOrange staining demonstrated that ox-LDL dramatically increased intracellular iron in THP-1 cells (Figure 1G,J,H,K), along with a substantial elevation in intracellular ROS stained with DCFH-DA (Figure 1M,N). Consistent results were observed by measuring iron and ROS levels using a microplate reader (Figure 1L,O). TEM revealed that ox-LDL treatment resulted in reduced mitochondrial size, increased mitochondrial membrane density, and fewer cristae in THP1 cells (Figure 1P,Q). Meanwhile, flow cytometry analysis showed that ox-LDL increased the fluorescence intensity of the lipid peroxidation reporter C11-BODIPY in THP-1 macrophages (Figure 1R,S). Furthermore, CCK-8 assay showed that ox-LDL significantly inhibited THP-1 cell proliferation (Figure 1I). Intriguingly, fer-1 effectively reversed all the effects induced by ox-LDL (Figure 1). Taken together, these data suggest that ox-LDL induces macrophage ferroptosis.

### 3.3. Knockdown of NEAT1 Ameliorates ox-LDL-Induced Ferroptosis in THP-1 Cells

**Figure 2:**
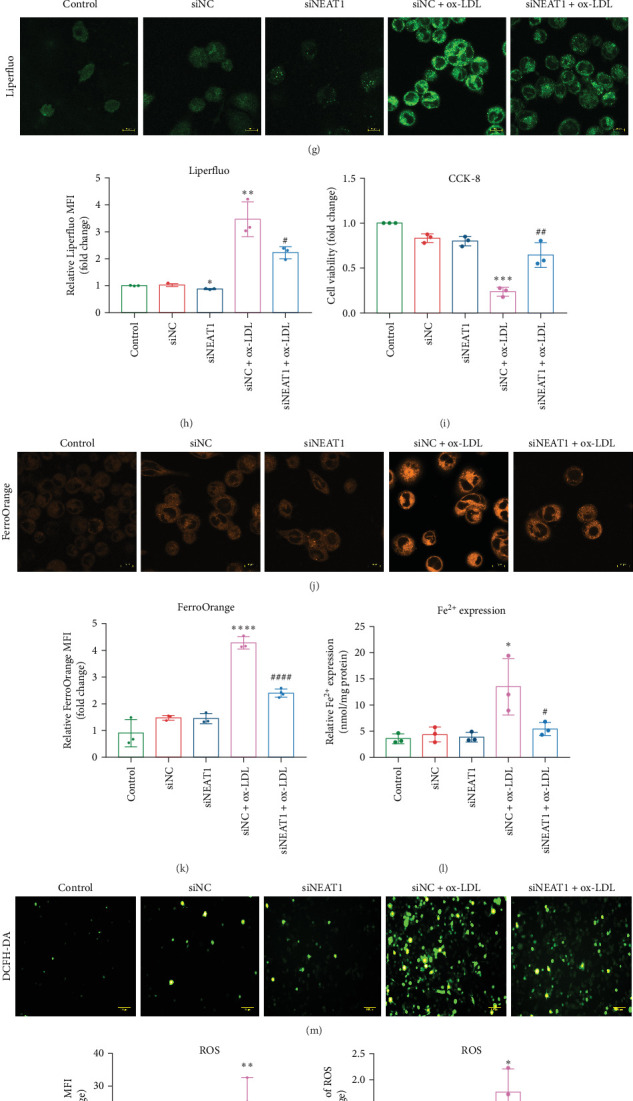
Knockdown of NEAT1 ameliorated ox-LDL-induced ferroptosis in THP-1 cells. THP-1 cells were transfected with small interfering RNA against NEAT1 (siNEAT1; 100 nM) or negative control (siNC) and incubated for 24 h, followed by ox-LDL treatment (100 µg/mL, 24 h). (A) NEAT1 expression was measured by qRT-PCR (*n* = 4). (B–F) STAT3 (B), pSTAT3 (C), ACSL4 (D), and GPX4 (E) protein expression and quantitative analysis determined by Western blot analysis (*n* = 3). (G) Intracellular lipid peroxidation detected by LiperFluo. Scale bar = 10 µm. (H) Quantitative analysis of LiperFluo. (I) CCK-8 assay (*n* = 3). (J) Intracellular iron detected by FerroOrange. Scale bar = 10 µm. (K) Quantitative analysis of FerroOrange. (L) An iron colorimetric assay kit was used to measure intracellular iron content at 550 nm using a microplate reader. (M) Intracellular reactive oxygen species (ROS) were detected by DCFH-DA. Scale bar = 100 µm. (N) Quantitative analysis of DCFH-DA. (O) A ROS assay kit was used to measure intracellular ROS levels at 488 mm and 525 mm using a microplate reader. (P) TEM analysis. Arrows indicate mitochondria. Scale bar = 0.5 µm. (Q) Quantitative analysis of TEM. Data are expressed as the mean ± SD. *⁣*^*∗*^*p* < 0.05, *⁣*^*∗∗*^*p* < 0.01 vs.siNC; #*p* < 0.05, ##*p* < 0.01 vs. siNC+ox-LDL.

Based on the above findings, we hypothesize that ox-LDL induces ferroptosis in THP-1 macrophages through mechanisms that may involve NEAT1 upregulation. To investigate the role of NEAT1 in macrophage ferroptosis, we silenced NEAT1 expression in THP-1 macrophages (Figure 2A). Knockdown of NEAT1 significantly decreased ASCL4 mRNA and protein expression (Figure 2B,C) while enhancing GPX4 expression (Figure 2B,D), regardless of ox-LDL presence. Moreover, Liperfluo and FerroOrange staining revealed that the knockdown of NEAT1 effectively abrogated the ox-LDL-induced increase in intracellular iron (Figure 2G,H,J,K) and ROS (Figure 2M,N) in THP-1 cells. Consistent results were observed by measuring iron and ROS levels using a microplate reader (Figure 2L,O). In the CCK-8 assay, the knockdown of NEAT1 reversed the loss of viability of THP-1 cells induced by ox-LDL (Figure 2I), along with the improvement of mitochondrial abnormalities (Figure 2P,Q). Given the role of NEAT1 in STAT3 regulation [17–19], we investigated the underlying mechanism in terms of STAT3 activation. Despite nonsignificant changes in total STAT3 (t-STAT3) mRNA (Figure S2C) and protein expression (Figure 2B,E), ox-LDL treatment significantly increased pSTAT3 protein level in THP-1 cells. Of note, knockdown of NEAT1 reduced STAT3 phosphorylation and reversed ox-LDL-induced upregulation of STAT3 phosphorylation (Figure 2B,F). These data collectively suggest that NEAT1 plays an essential role in macrophage ferroptosis in AS development, and the mechanism involves STAT3 activation.

### 3.4. NEAT1 Promotes THP-1 Cell Ferroptosis Through STAT3 Phosphorylation

Considering the established interaction between NEAT1 and STAT3 and the role of STAT3 in promoting ferroptosis and oxidative stress [30, 31], we sought to elucidate their association in AS. In PBMCs from CHD patients, STAT3 expression was significantly correlated with NEAT1 expression (*r* = 0.4102, *p* < 0.0001; Figure S3E), iron content (*r* = 0.3376, *p* < 0.0001; Figure S3F), and Gensini scores (*r* = 0.3469, *p* < 0.0001; Figure S3G). These data suggest a pivotal role of NEAT1 and STAT3 in the progression of AS.

**Figure 3:**
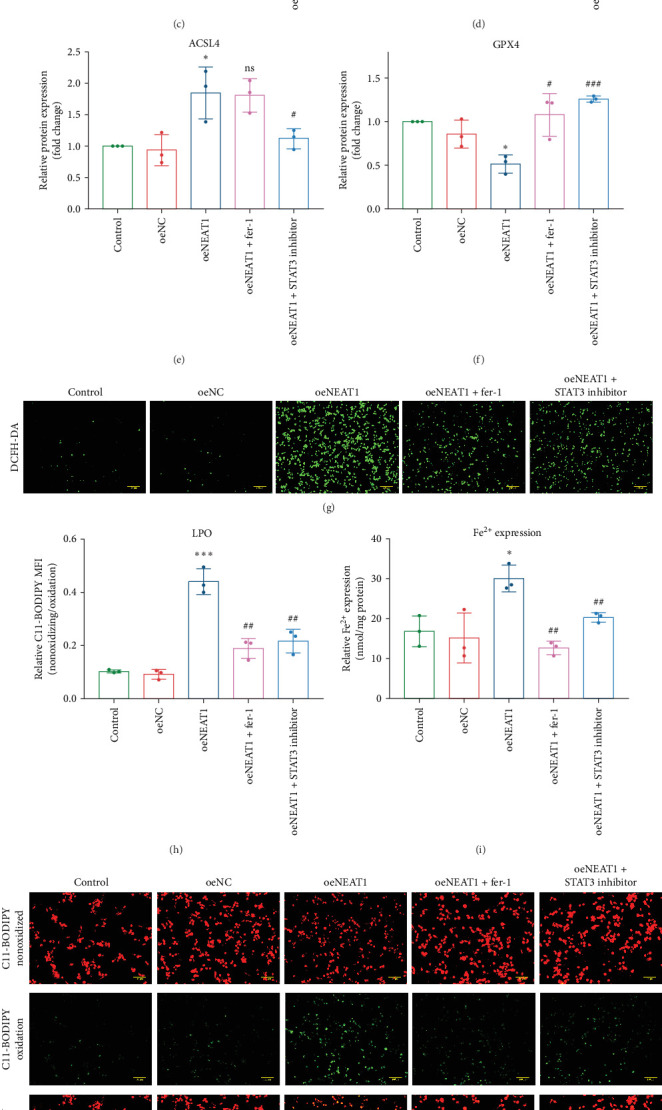
NEAT1 promoted ferroptosis of THP-1 cells through STAT3 phosphorylation. (A) THP-1 cells were transfected with NEAT1-overexpressing vectors. qRT-PCR was performed to determine NEAT1 expression (*n* = 3). (B–F) STAT3, pSTAT3, ACSL4, and GPX4 were determined by Western blot analysis and quantitative analysis (*n* = 3). (G) Intracellular ROS detected by DCFH-DA. Scale bar = 100 µm. (H) Quantitative analysis of DCFH-DA. (I) Intracellular iron level (*n* = 3). (J) Intracellular lipid peroxidation detected by C11-BODIPY. Scale bar = 100 µm. (K) Quantitative analysis of C11-BODIPY. Data are expressed as the mean ± SD. *⁣*^*∗*^*p* < 0.05, *⁣*^*∗∗∗*^*p* < 0.001, *⁣*^*∗∗∗∗*^*p* < 0.0001 vs. control; #*p* < 0.05, ###*p* < 0.001 vs. ox-LDL; ns, nonsignificant.

Next, we investigated whether NEAT1 contributes to ferroptosis through STAT3 activation. We found that overexpression of NEAT1 (Figure 3A) markedly enhanced pSTAT3 protein expression without affecting t-STAT3 expression (Figure 3B–D), accompanied by significant upregulation of ACSL4 expression and downregulation of GPX4 expression (Figure 3B,E,F) in THP-1 cells. After optimizing the concentration and duration of STAT3 inhibitor treatment (Figure S3A–D), we found that the STAT3 inhibitor significantly reversed the effect of NEAT1 overexpression on pSTAT3, ACSL4, and GPX4 expression, similar to ferroptosis inhibitor fer-1 (Figure 3B–F). Furthermore, the overexpression of NEAT1 dramatically increased ROS production in THP-1 cells, as demonstrated by DCFH-DA (Figure 3G,K) and C11-BODIPY (Figure 3J,H) staining, which was partly but significantly reversed by fer-1 or STAT3 inhibitor. Similar results were observed in intracellular iron content (Figure 3I). These data suggest that NEAT1 promotes macrophage ferroptosis through STAT3 activation in AS.

## 4. Knockdown of NEAT1 Promotes Ubiquitination of pSTAT3

**Figure 4:**
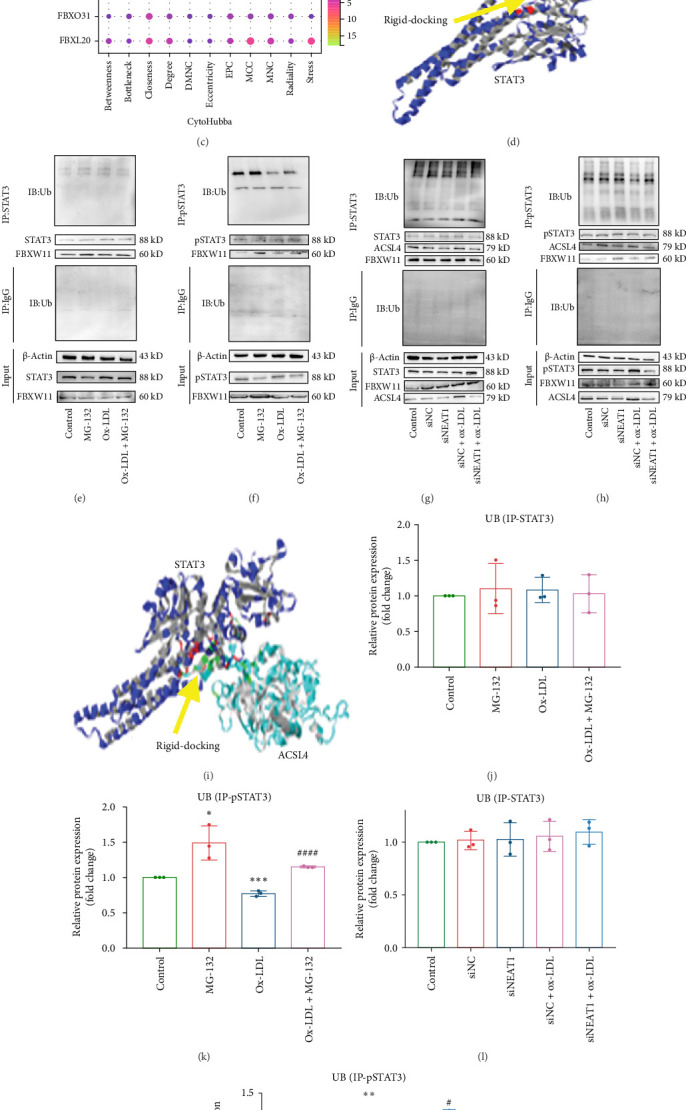
Physical interaction of NEAT1-STAT3, identification of downstream molecules, and pSTAT3 ubiquitination regulated by NEAT1. (A) RNA binding protein immunoprecipitation (RIP) was performed to identify the physical interaction between NEAT1 and STAT3. (B) Venn diagram showing the overlap genes that potentially bind to STAT3 in iUUCD and ChIPBase databases. (C) Hub genes. (D) Rigid docking of STAT3 and FBXW11. (E, F) THP-1 cells were treated with ox-LDL (100 µg/mL, 24 h) and MG-132 (10 µM, 6 h), alone or in combination. Immunoprecipitation (IP) was conducted to examine the ubiquitination of STAT3. (F) and pSTAT3 (Y705). (G, H) NEAT1 expression was silenced in THP-1 cells, followed by treatment with ox-LDL (100 µg/mL, 24 h), siNC, and siNEAT1, alone or in combination. IP was conducted to examine the ubiquitination of STAT3 (H) and pSTAT3 (Y705). (I) Rigid docking of STAT3 and ACSL4. (J–M) Quantitative analysis of protein expression of Ub was determined (*n* = 3). Data are expressed as the mean ± SD. *⁣*^*∗*^*p* < 0.05, *⁣*^*∗∗∗*^*p* < 0.001, *⁣*^*∗∗∗∗*^*p* < 0.0001 vs. siNC; #*p* < 0.05, ###*p* < 0.001 vs. ox-LDL or siNC+ox-LDL. ns, nonsignificant.

It has been reported that STAT3 can bind to the NEAT1 promoter [32]; therefore, we examined the physical interaction between NEAT1 and STAT3 in THP1 cells. The results of the RIP assay suggest that NEAT1 and STAT3 physically interacted (Figure 4A). Because NETA1 has been shown to regulate the ubiquitination of STAT family proteins [17, 18], we searched Chipbase and iUUCD for potential STAT3-binding proteins and found nine candidate proteins shared by the two databases (Figure 4B). Ubiquitin ligase FBXW11 is the hub protein in the PPI network (Figure 4C), suggesting that NEAT1 binds to STAT3 and triggers STAT3 ubiquitination through FBXW11.

To verify the physical association between STAT3 and FBXW11, and to explore ACSL4's involvement in this regulatory network influenced by NEAT1, we constructed 3D structures of STAT3, FBXW11, and ACSL4 (Figure S4I–N). The Ramachandran plot showed that the most favorable and allowable zone was 91.4% for STAT3, 92.5% for FBXW11, and 90.4% for ACSL4 (Figure S4L–N). ProSa analysis demonstrated that the Z-scores of STAT3, FBXW11, and ACSL4 were −11.24, −9.73, and −6.53, respectively, within the range of the natively folded protein (Figure S4L–N). Then, we selected the STAT3-FBXW11 and STAT3-ACSL4 docking models with the lowest binding energy (Figure 4D,I). Co-IP analysis confirmed that STAT3 and pSTAT3 physically bound to FBXW11 and ACSL4 (Figure S4A–H). These results suggest that NEAT1 modulates macrophage ferroptosis through the ubiquitination of STAT3, mediated by interactions with FBXW11 and ACSL4 in the NEAT1/STAT3 signaling pathway.

Based on the above findings, we investigated whether NEAT1 modulates STAT3 ubiquitination in ox-LDL-induced THP-1 cells. We found that ox-LDL and proteasome inhibitor MG-132, alone or in combination, did not affect the ubiquitination of t-STAT3 in THP-1 cells (Figure 4E,J). However, ox-LDL significantly reduced ubiquitinated pSTAT3 expression, while MG-132 had the opposite effect. Ox-LDL also inhibited the promotion of pSTAT3 ubiquitination by MG-132 (Figure 4F,K). Importantly, without affecting STAT3 ubiquitination (Figure 4G,L), knockdown of NEAT1 significantly promoted pSTAT3 ubiquitination and reversed the inhibitory effect of ox-LDL on pSTAT3 ubiquitination (Figure 4H,M). In contrast to ubiquitinated pSTAT3, ACSL4 protein expression showed an opposite pattern, while FBXW11 protein expression showed a similar pattern (Figure 4G,H). These results suggest that NEAT1 selectively regulates the ubiquitination of pSTAT3, thereby influencing ox-LDL-induced ferroptosis in THP-1 cells through a mechanism that involves modulating the activity of FBXW11 and inversely affecting ACSL4 expression.

### 4.1. NEAT1 Depletion Prevents HFD-Induced AS and Ferroptosis

**Figure 5:**
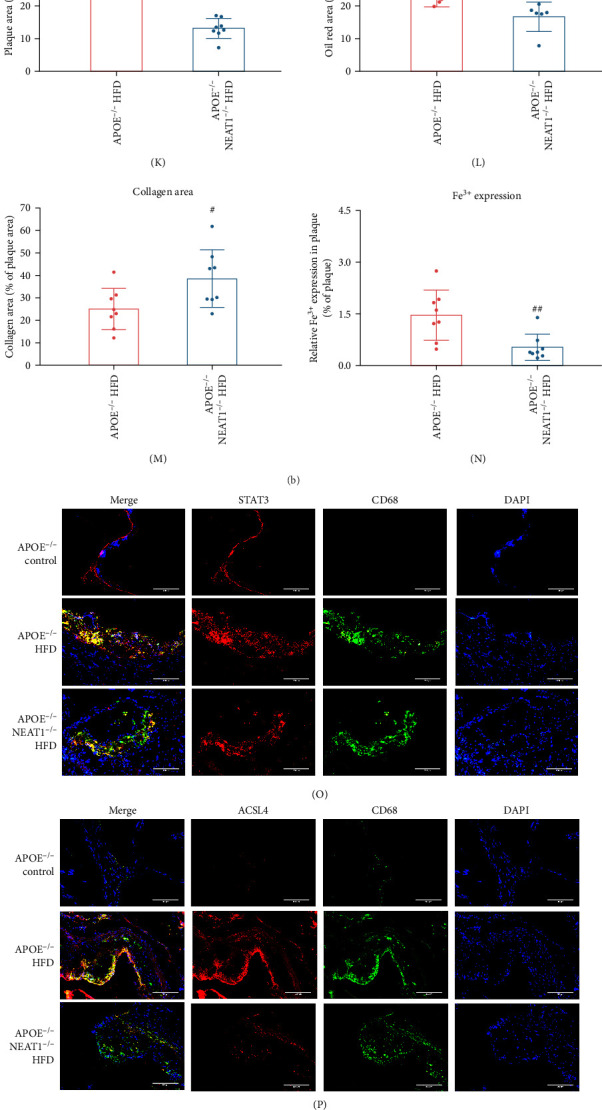
NEAT1 deficiency prevented high-fat diet (HFD)-induced ferroptosis and atherosclerosis in APOE^−/−^ mice. 8-week-old APOE^−/−^ and APOE^−/−^NEAT^−/−^ mice were fed an HFD for 16 weeks. APOE^−/−^ mice were given a chow diet (*n* = 8 per group). (A) Fluorescence in situ hybridization (FISH) was conducted to detect NEAT1 expression in aortic atherosclerotic plaques. (B) Dihydroethidium (DHE) staining was performed to detect arterial ROS. Scale bar = 200 µm. (C) Hematoxylin and eosin (HE) staining. Scale bar = 200 µm. (D) Oil Red O staining. Scale bar = 200 µm. (E) Masson staining. Scale bar = 200 µm. (F) Prussian blue staining. Scale bar = 200 µm. (G) qRT-PCR was performed to determine NEAT1 expression in PBMCs (*n* = 5). (H–J) ROS, MDA, and iron levels in the atherosclerotic plaques. (K) Quantitative analysis of HE staining. (L) Quantitative analysis of Oil Red O staining. (M) Quantitative analysis of Masson staining. (N) Quantitative analysis of Prussian blue staining. (O, P) Immunofluorescence analysis of STAT3 and ACSL4 in aortic atherosclerotic plaques. Scale bar = 200 µm. (Q, R) STAT3 and ACSL4 were determined by Western blot analysis and quantitative analysis (*n* = 3). (S, T) Immunofluorescence analysis of pSTAT3 and GPX4 in aortic atherosclerotic plaques. Scale bar = 200 µm. (U, V) pSTAT3 and GPX4 were determined by Western blot analysis and quantitative analysis (*n* = 3). Data are expressed as the mean ± SD. *⁣*^*∗∗*^*p* < 0.01, *⁣*^*∗∗∗*^*p* < 0.001 vs. APOE^−/−^ control; #*p* < 0.05 vs. APOE^−/−^ + HFD.

To investigate the role of NEAT1 in ferroptosis and AS in vivo, we fed APOE^−/−^ and APOE^−/−^NEAT1^−/−^ mice HFD for 16 weeks to induce AS. FISH (Figure 5A) and qRT-PCR (Figure 5G) confirmed NEAT1 depletion in aortic tissue samples and PBMCs from APOE^−/−^NEAT1^−/−^ mice. HFD significantly upregulated NEAT1 expression in atherosclerotic plaques, along with an increase in pSTAT3 and ACSL4 staining and a decrease in GPX4 staining in macrophages. NEAT1 deletion eliminated these effects (Figure 5O–T). Consistent results were observed in Western blot analysis (Figure 5Q–V) as well as STAT3, ACSL4, and GPX4 mRNA expression in PBMCs (Figure S5A–C). In addition, NEAT1 deficiency abolished HFD-induced ROS accumulation in atherosclerotic plaques stained with DHE (Figure 5B). Consistently, in plasma (Figure S5D–F) and aortic atherosclerotic plaques (Figure 5H–J), NEAT1 deficiency blocked HFD-induced elevations in ROS, MDA, and iron levels. Histological studies including Prussian blue staining, HE staining, Masson staining, and Oil Red O staining showed that knockout of NEAT1 greatly reduced HFD-induced iron accumulation (Figure 5F,N), atherosclerotic plaque burden (Figure 5C,K), collagen production (Figure 5E,M), and lipid deposition (Figure 5D,L) in aortic plaques. Taken together, these results suggest that knockdown of NEAT1 prevents HFD-induced macrophage ferroptosis and AS.

### 4.2. Exercise Reduces NEAT1 Expression and Prevents HFD-Induced Ferroptosis and AS

**Figure 6:**
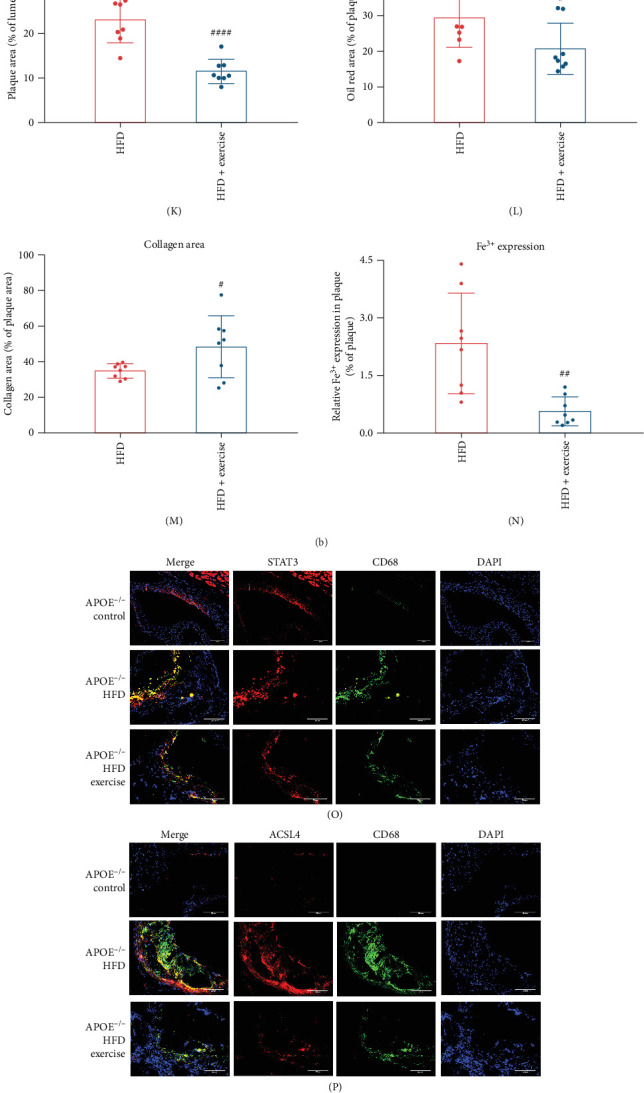
Exercise reduced NEAT1 expression and prevented HFD-induced ferroptosis and atherosclerosis in mice. 8-week-old APOE^−/−^ and APOE^−/−^NEAT^−/−^ mice were fed an HFD for 16 weeks. APOE^−/−^ mice were given a chow diet (*n* = 8 per group). (A) Fluorescence in situ hybridization (FISH) was conducted to detect NEAT1 expression in aortic atherosclerotic plaques. (B) Dihydroethidium (DHE) staining was performed to detect arterial ROS. Scale bar = 200 µm. (C) Hematoxylin and eosin (HE) staining. Scale bar = 200 µm. (D) Masson staining. Scale bar = 200 µm. (E) Oil Red O staining. Scale bar = 200 µm. (F) Prussian blue staining. Scale bar = 200 µm. (G) qRT-PCR was performed to determine NEAT1 expression in PBMCs (*n* = 5). (H–J) ROS, MDA, and iron levels in the atherosclerotic plaques. (K) Quantitative analysis of HE staining. (L) Quantitative analysis of Oil Red O staining. (M) Quantitative analysis of Masson staining. (N) Quantitative analysis of Prussian blue staining. (O, P) Immunofluorescence analysis of STAT3 and ACSL4 in aortic atherosclerotic plaques. Scale bar = 200 µm. (Q, R) STAT3 and ACSL4 were determined by Western blot analysis and quantitative analysis (*n* = 3). (S, T) Immunofluorescence analysis of pSTAT3 and GPX4 in aortic atherosclerotic plaques. Scale bar = 200 µm. (U, V) pSTAT3 and GPX4 were determined by Western blot analysis and quantitative analysis (*n* = 3). Data are expressed as the mean ± SD. *⁣*^*∗*^*p* < 0.05, *⁣*^*∗∗*^*p* < 0.01, *⁣*^*∗∗∗*^*p* < 0.001, *⁣*^*∗∗∗∗*^*p* < 0.0001 vs. APOE^−/−^ control; #*p* < 0.05, ##*p* < 0.01, ###*p* < 0.001 vs. APOE^−/−^ + HFD.

Given that LncRNAs are potential therapeutic targets for cardiovascular diseases in physical exercise [33], we wondered whether exercise targets NEAT1 expression. FISH assay showed that exercise suppressed NEAT1 expression in aortic tissue (Figure 6A) and PBMCs (Figure 6G) of HFD-fed ApoE^−/−^ mice. Exercise suppressed STAT3 and ACSL4 mRNA expression while promoting GPX4 mRNA expression in mouse PBMCs (Figure S6A–C), along with decreases in ROS, MDA, and iron levels in plasma (Figure S6D–F). Exercise training exhibited the same effects as NEAT1 knockout on ferroptosis and AS indicators, including attenuating pSTAT3 expression without changing STAT3 expression (Figure 6O,S,Q,U and Figure S6I,J), reducing ACSL4 expression (Figure 6P,R and Figure S6B,K), enhancing GPX4 expression (Figure 6T and Figure S6L), reducing ROS, MDA, and iron levels in both plasma (Figure S6P–R) and plaques (Figure 6H–J and Figure S6M–O), as well as alleviating histological abnormalities in aortic plaques of HFD-fed mice (Figure 6C–F, K–N). Taken together, these data suggest that NEAT1 may be a promising therapeutic target for preventing macrophage ferroptosis and AS.

## 5. Exercise Reduces NEAT1 and Ferroptosis Indicators in PBMCs From Patients With CHD

**Figure 7:**
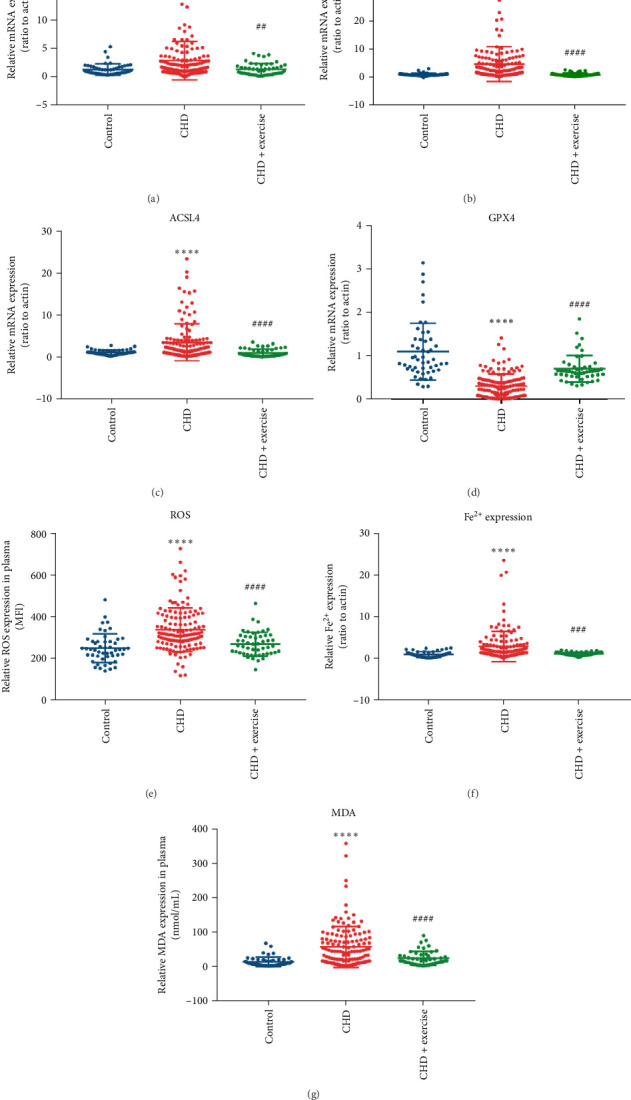
Exercise reduced plasma NEAT1 and ferroptosis indicators in patients with CHD. PBMCs were obtained from healthy individuals (*n* = 50), CHD patients with regular exercise (*n* = 50), and CHD patients without regular exercise (*n* = 84). (A–D) NEAT1, STAT3, ACSL4, and GPX4 expression detected by qRT-PCR. (E–G) Plasma ROS, iron, and MDA levels. Data are expressed as the mean ± SD. *⁣*^*∗∗∗*^*p* < 0.001, *⁣*^*∗∗∗∗*^*p* < 0.0001 vs. control; ##*p* < 0.01, ###*p* < 0.001, ####*p* < 0.0001 vs. CHD.

To investigate the clinical significance of our findings, we compared the expression of NEAT1 and ferroptosis indicators in PBMCs and plasma among healthy individuals (*n* = 50), CHD patients with regular exercise (*n* = 50), and patients without regular exercise (*n* = 84). Notably, baseline total cholesterol (TC) was significantly higher in the CHD + exercise group than in the CHD group (5.18 ± 1.64 vs. 4.28 ± 1.01 mmol/L, *p*=0.011; Table S1). The results of qRT-PCR revealed that NEAT1 levels were significantly higher in PBMCs from CHD patients without exercise than in patients who exercised regularly (Figure 7A). Similar trends were observed in STAT3 and ACSL4 levels in PBMCs as well as OS, MDA, and iron levels in plasma, except for GPX4 (Figure 7B–G). These findings suggest that exercise could be used to treat CHD by targeting NEAT1 and ferroptosis.

To further support the mechanistic role of NEAT1 in regulating pSTAT3, we conducted additional validation experiments. FISH confirmed that NEAT1 was primarily localized in the nucleus of THP-1 cells (Figure S7A), consistent with its role in transcriptional regulation and previously reported nuclear enrichment [34]. qRT-PCR validated the efficiency of NEAT1 knockdown and overexpression (Figure S7B). Co-IP assays demonstrated that NEAT1 specifically modulates the ubiquitination of pSTAT3 but not t-STAT3 (Figure S7C–E), aligning with our earlier findings and further substantiating that NEAT1 selectively affects pSTAT3 stability.

## 6. Discussion

This study demonstrated for the first time that NEAT1 promotes macrophage ferroptosis in AS development, aligning with findings by Zhang et al. [35], which described the role of NEAT1 in promoting ferroptosis in hepatocellular carcinoma. Our results provide new insights into this area of research by revealing that knockdown of NEAT1 inhibited ferroptosis by suppressing STAT3 activation possibly through promoting pSTAT3 ubiquitination. NEAT1 deletion alleviated HFD-induced macrophage ferroptosis and AS in APOE^−/−^ mice. Aerobic exercise blocked HFD-induced NEAT1 upregulation in macrophages and ameliorated HFD-induced macrophage ferroptosis and AS in mice. Targeting NEAT1 and NEAT1-induced macrophage ferroptosis is a promising therapeutic approach to preventing and treating AS.

NEAT1 contributes to paraspeckle formation, transcriptional activation, and inflammation regulation under physiological and pathological conditions [36]. Emerging evidence highlights NEAT1 as a critical regulator in AS pathogenesis. Silencing NEAT1 has been shown to inhibit vascular smooth muscle cell proliferation, migration, and osteogenic differentiation by disrupting EZH2-mediated epigenetic regulation, suggesting that NEAT1 inhibition may suppress plaque progression [37]. Similarly, targeting NEAT1 reduces human aortic endothelial cell proliferation and promotes apoptosis through the miR-638/PGK1/AKT-mTOR signaling pathway [38]. In addition, NEAT1 knockdown mitigates ox-LDL-induced inflammation, oxidative stress, foam cell formation, and apoptosis inhibition by regulating miR-128 [39]. NEAT1 also exerts pro-inflammatory effects in vascular endothelial cells, with its inhibition attenuating TNF-α-induced upregulation of inflammatory mediators such as CXCL8, CCL2, VCAM1, and ICAM1 [40]. In macrophages, NEAT1 silencing suppresses lipid uptake and inflammation by modulating miR-342-3 p, further highlighting its role in inflammation regulation during AS [7]. Our previous studies have shown that NEAT1 induces immune tolerance, and NEAT1-silenced dendritic cells reduce inflammatory cell infiltration in mouse models [41]. Clinically, NEAT1 is significantly upregulated in PBMCs and AS plaques derived from patients with coronary artery disease [40]. In line with this, our study found that plasma NEAT1 levels were positively correlated with disease severity in patients with CHD. Moreover, we identified that NEAT1 promotes macrophage ferroptosis during AS development, introducing a novel mechanism of its involvement in AS and providing a potential therapeutic target for AS management.

Ferroptosis is positively correlated with AS severity [13, 40, 42, 43], suggesting its critical role in disease progression. NEAT1 has been implicated in regulating ferroptosis in various diseases, including cardiovascular conditions. In acute myocardial infarction, silencing NEAT1 alleviates disease severity by suppressing ferroptosis via the miR-450 b-5 p/ACSL4 pathway [44]. Similarly, in ischemic stroke, NEAT1 promotes ferroptosis by acting as a ceRNA, sponging miR-17-5p, and enhancing TLR4 expression [45]. NEAT1 also regulates ferroptosis in cancer and neurological disorders. It exacerbates ferroptosis in hepatocellular carcinoma by modulating the miR-362-3 p/MIOX axis [35], contributes to the ferroptosis-enriched microenvironment in glioma [15], and serves as a therapeutic target for ferroptosis induction in non-small-cell lung cancer [46]. Furthermore, NEAT1 promotes ferroptosis in Parkinson's disease [47], *Streptococcus* pneumoniae-induced alveolar epithelial cell injury [48], and sepsis-associated encephalopathy [16] by modulating different molecular axes. Thus, we speculated that NEAT1 might contribute to AS by regulating macrophage ferroptosis. Our study demonstrated iron overload in the plasma of CHD patients and ox-LDL-induced ferroptosis in THP-1 macrophages. Both plasma NEAT1 and iron levels were positively correlated with the severity of CHD. These findings suggest that NEAT1 is closely associated with ferroptosis in AS development.

In THP-1 macrophages, we found that NEAT1 physically interacted with STAT3 and promoted ferroptosis by phosphorylating STAT3. NEAT1 has been shown to promote Th2 cell differentiation by suppressing STAT6 ubiquitination [18]. Similarly, NEAT1 inhibits SIRT6 ubiquitination, promoting inflammation in rheumatoid arthritis [49]. Furthermore, NEAT1 translocated from the nucleus to the cytoplasm interacts with E3 ubiquitin ligase to promote DVL2 degradation, acting as a tumor suppressor in acute myeloid leukemia [50]. Thus, NEAT1 may regulate the expression of protein targets through ubiquitination. Indeed, our results showed that knockdown of NEAT1 promoted pSTAT3 ubiquitination, leading to the downregulation of pSTAT3 expression and subsequent alleviation of ox-LDL-induced ferroptosis in THP-1 cells. Notably, prior studies have shown that STAT3 can transcriptionally activate NEAT1 by binding to its promoter region [51]. Together with our RIP results showing physical interaction between NEAT1 RNA and STAT3 protein, these findings suggest a bidirectional regulatory loop in which STAT3 promotes NEAT1 expression, and NEAT1 in turn stabilizes STAT3 signaling by limiting pSTAT3 degradation.

We also found that NEAT1 promoted ferroptosis in THP-1 cells through STAT3 phosphorylation. pSTAT3 promotes AS development [52] and plays an important role in ferroptosis and oxidative stress response [30, 31, 53]. Moreover, STAT3 interacts with ubiquitin ligase FBXW7, facilitating proteasomal ubiquitination degradation [54]. In acute lymphoblastic leukemia, FBXW7 mediates VDAC3 ubiquitination, resulting in ferroptosis [24]. Intriguingly, our study identified FBXW11 and the ferroptosis marker ACSL4 as downstream effectors of NEAT1/STAT3 signaling in THP-1 cells, suggesting that FBXW11 regulates macrophage ferroptosis in AS. Our Co-IP data suggest that NEAT1 knockdown increases the ubiquitination of pSTAT3 but not total STAT3. However, whether FBXW11 directly influences STAT3 phosphorylation or only promotes degradation of pSTAT3 remains to be fully elucidated. FBXW11 may influence STAT3 activation indirectly by regulating pSTAT3 turnover and stability rather than interfering with its phosphorylation. There are currently no studies reporting the involvement of FBXW11 in AS or its connection to STAT3 degradation and phosphorylation. Although pSTAT3 and t-STAT3 differ only by a phosphate group, phosphorylation can induce conformational changes that expose lysine residues or generate recognition motifs for specific E3 ligases [55], such as FBXW11. This may explain why NEAT1 knockdown selectively enhances pSTAT3 ubiquitination, while total or unphosphorylated STAT3 remains unaffected. In addition, since t-STAT3 reflects the overall pool of both phosphorylated and unphosphorylated forms, selective degradation of pSTAT3 may not substantially reduce t-STAT3 protein levels. Taken together, our findings reveal a previously uncharacterized mechanism by which NEAT1 contributes to AS. Future studies should explore whether targeting NEAT1 represents a promising therapeutic strategy, such as RNA interference, antisense oligonucleotides, or nanoparticle-based delivery systems.

Our cell and animal studies suggest that NEAT1 is a potential therapeutic target for AS management. Given that aerobic exercise has been shown to prevent AS and cardiovascular disease by targeting lncRNAs [33], we investigated whether exercise could regulate NEAT1 expression and macrophage ferroptosis in AS. Consistent with previous studies, our results showed that aerobic exercise reduced plasma and plaque NEAT1 expression and alleviated HFD-induced macrophage ferroptosis and AS in mice [56]. This highlights the therapeutic potential of lifestyle interventions in AS management by modulating lncRNA expression. Similarly, exercise attenuated the expression of NEAT1 and ferroptosis indicators in the plasma of CHD patients, supporting the hypothesis that exercise, as a nonpharmacological strategy, may prevent AS by targeting NEAT1 expression and macrophage ferroptosis. Notably, baseline TC was significantly higher in the CHD + exercise group than in the CHD group. This unexpected finding may be attributed to suboptimal exercise intensity or frequency, insufficient adherence, or postexercise dietary compensation, which could counteract the lipid-lowering effects of physical activity despite molecular improvements.

Although this study focused on macrophage ferroptosis, we acknowledge that global NEAT1 knockout may influence other vascular cell types. Prior studies have shown that NEAT1 promotes endothelial pyroptosis and vascular inflammation through KLF4/NLRP3 signaling, and that exercise downregulates NEAT1 to attenuate endothelial dysfunction in AS [56]. NEAT1 has also been implicated in vascular smooth muscle cell differentiation and calcification [37]. These findings suggest that NEAT1 may exert multifaceted effects across vascular compartments. Our future work will evaluate the role of NEAT1 in nonmacrophage cell types to better delineate its systemic contribution to atherogenesis.

This study has some limitations. First, the association between exercise-induced NEAT1 downregulation and disease severity of CHD patients remains uninvestigated due to a lack of angiographic results postexercise. Second, confounding factors such as age and gender were not included in this study when analyzing correlations due to scope, timeline, and resource constraints. Third, re-expressing NEAT1 in APOE^−/−^NEAT1^−/−^ mice is required to determine if exercise prevents AS by targeting NEAT1. In addition, all participants were of Han ethnicity in China, which may limit the generalizability of the findings to other populations, and the relatively small sample size may reduce statistical power. Finally, further research is needed on the role of FBXW11 in NEAT1/STAT3-regulated macrophage ferroptosis.

## 7. Conclusions

In conclusion, NEAT1 promotes macrophage ferroptosis in AS development by suppressing pSTAT3 ubiquitination. Targeting NEAT1 is a promising therapeutic approach against AS and subsequent CHD.

## Figures and Tables

**Table 1:** Gensini scoring system.

Stenosis	Score	Lesion	Score
1%–25%	1	LM	5
26%–50%	2	Proximal LAD or LCX	2.5
51%–75%	4	Middle LAD	1.5
76%–90%	8	Distal LAD	1.0
91%–99%	16	Middle or distal LCX	1.0
100%	32	RCA	1.0
		Small branch	0.5

Abbreviations: LAD, left interior descend; LCX, left circumflex; LM, left main; RCA, right coronary artery.

## Data Availability

The datasets generated and/or analyzed during the current study are available from the corresponding author upon reasonable request.

## Nomenclature

CHD: Coronary heart disease
AS: Atherosclerosis
NEAT1: Nuclear enriched abundant transcript 1
ROS: Reactive oxygen species
HFD: High-fat diet
PBMCs: Peripheral blood mononuclear cells
HE: Hematoxylin and eosin
qRT-PCR: Quantitative real-time PCR
DCFH-DA: Dichlorodihydrofluorescein diacetate
MDA: Measurement of malondialdehyde
RIP: RNA binding protein immunoprecipitation
Co-IP: Co-immunoprecipitation
FISH: Fluorescence in situ hybridization
CCK-8: Cell counting kit-8
TEM: Transmission electron microscope
PPI: Protein–protein interaction.

## Supporting Information

Supporting Information Figure S1. Ox-LDL upregulated NEAT1 expression and induced ferroptosis in THP-1 macrophages. Figure S2. Knockdown of NEAT1 ameliorated ox-LDL-induced ferroptosis in THP-1 cells. Figure S3. Optimization of STAT3 inhibitor treatment. Figure S4. Co-IP analysis and identification of downstream molecules, and pSTAT3 ubiquitination regulated by NEAT1. Figure S5. NEAT1 deficiency prevented HFD-induced ferroptosis and atherosclerosis in APOE^−/−^ mice. Figure S6. Exercise reduced NEAT1 expression and prevented HFD-induced ferroptosis and atherosclerosis in mice. Figure S7. NEAT1 is predominantly nuclear and selectively regulates pSTAT3 ubiquitination. Table S1. Clinical characteristics of human subjects. Table S2. PCR primer sequences.
