# Supplementary material for: LncRNA NEAT1 Knockdown Alleviates Macrophage Ferroptosis and Atherosclerosis by Suppressing STAT3 Activation
Source: Mediators Inflamm. 2025 Nov 12;2025:8862449. doi: 10.1155/mi/8862449 (PMC12629693; doi:10.1155/mi/8862449)
Supplement: Supporting Information — Figure S1. Ox-LDL upregulated NEAT1 expression and induced ferroptosis in THP-1 macrophages. Figure S2. Knockdown of NEAT1 ameliorated ox-LDL-induced ferroptosis in THP-1 cells. Figure S3. Optimization of STAT3 inhibitor treatment. Figure S4. Co-IP analysis and identification of downstream molecules, and pSTAT3 ubiquitination regulated by NEAT1. Figure S5. NEAT1 deficiency prevented HFD-induced ferroptosis and atherosclerosis in APOE−/− mice. Figure S6. Exercise reduced NEAT1 expression and prevented HFD-induced ferroptosis and atherosclerosis in mice. Figure S7. NEAT1 is predominantly nuclear and selectively regulates pSTAT3 ubiquitination. Table S1. Clinical characteristics of human subjects. Table S2. PCR primer sequences. [file 8862449.f1.docx]

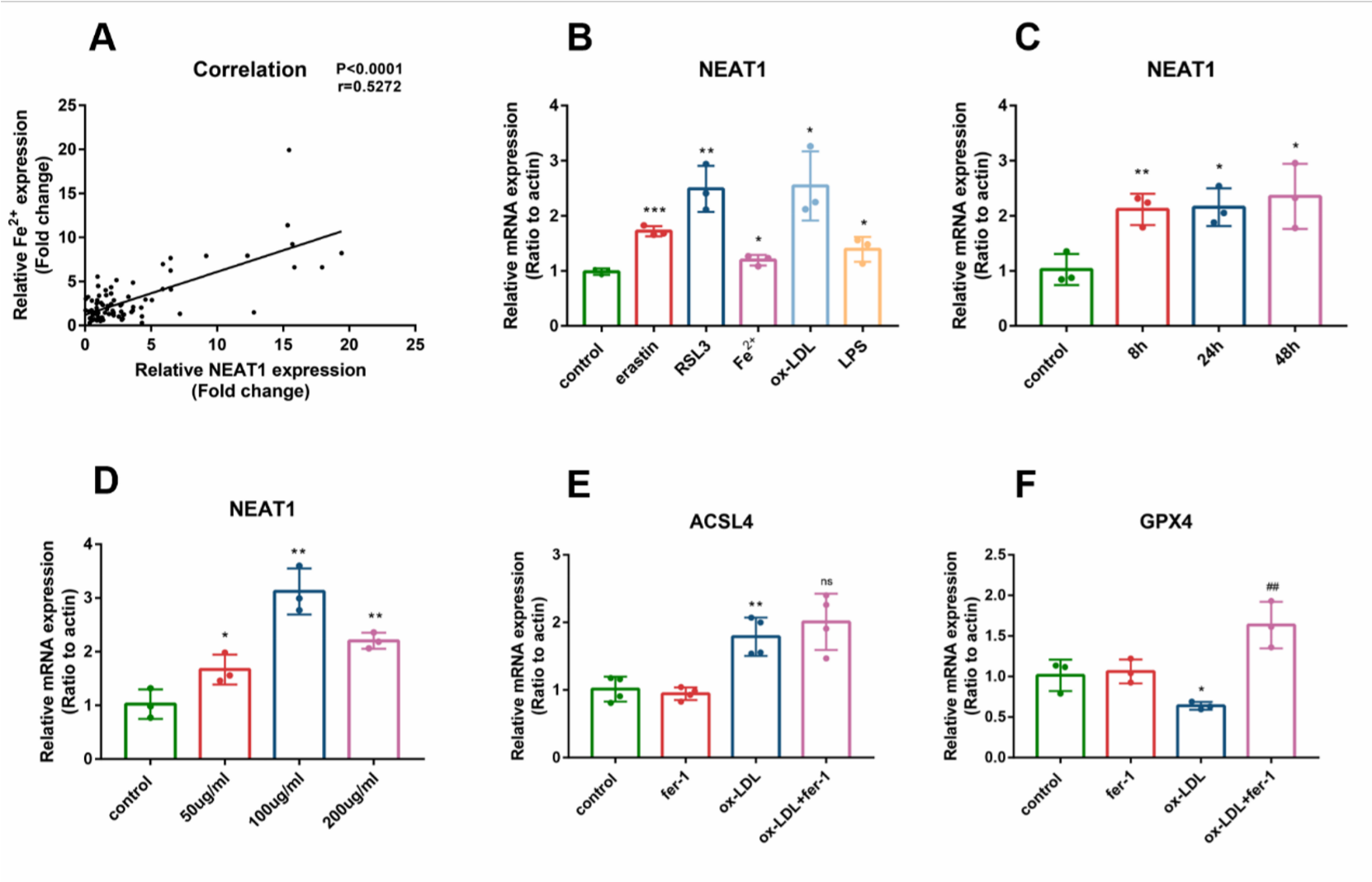


**Figure S1 Ox-LDL upregulated NEAT1 expression and induced ferroptosis in THP-1 macrophages.** (A) Correlation between PBMCs NEAT1 and plasma iron levels in CHD patients. (B) THP-1 cells were treated with ferroptosis inducers (erastin, RSL3, and iron) and atherosclerosis inducers (ox-LDL and LPS). qRT-PCR was performed to determine NEAT1 expression (n = 3). (C) THP-1 cells were treated with 50 µg/mL ox-LDL for 8 h, 24 h, or 48h. qRT-PCR was performed to determine NEAT1 expression (n = 3). (D) THP-1 cells were exposed to 50 µg/mL,100 µg/mL, or 200 µg/mL ox-LDL for 24 h. qRTPCR was performed to determine NEAT1 expression (n = 3). (E-F) mRNA expression of ACSL4 and GPX4 (n = 3). Data are expressed as the mean ± SD Data are expressed as the mean ± standard deviation (SD). **p* < 0.05, ***p* < 0.01, ****p* < 0.001, vs. control; #*p* < 0.05, ##*p* < 0.01, ###*p* < 0.001 vs. ox-LDL; ns, non-significant.


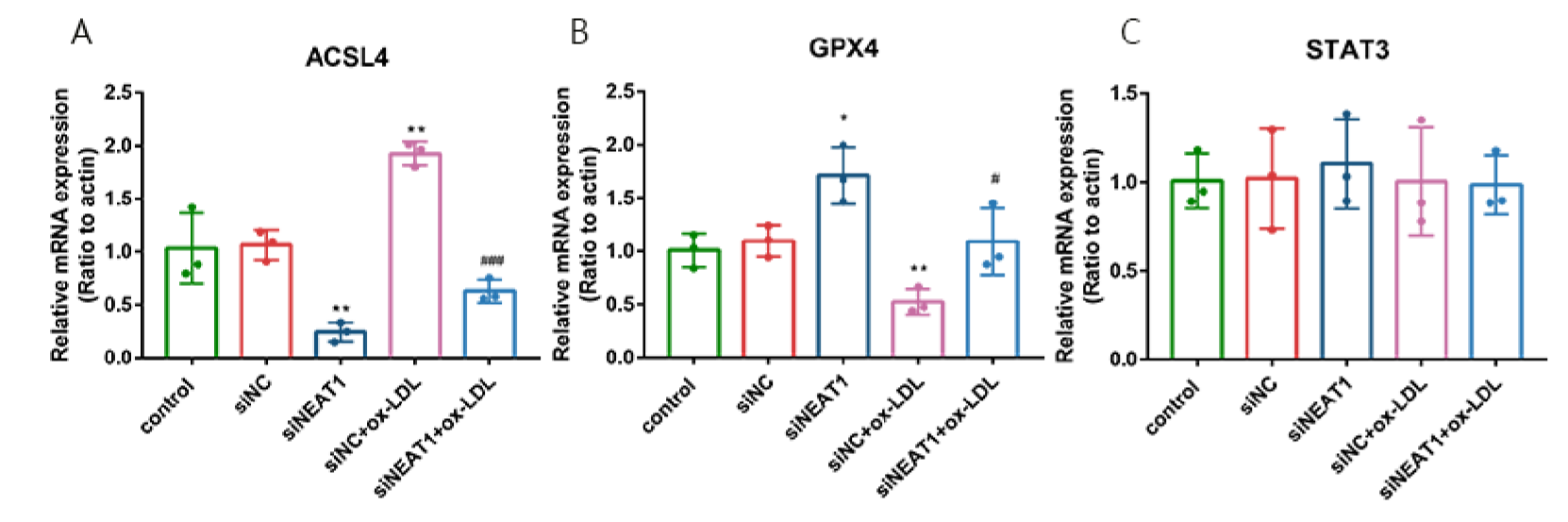


**Figure S2 Knockdown of NEAT1 ameliorated ox-LDL-induced ferroptosis in THP-1 cells.**

(A–C) ACSL4, GPX4 and STAT3 mRNA expression determined by qRT-PCR (n = 3). Data are expressed as the mean ± SD. **p* < 0.05, ***p* < 0.01, *****p* < 0.0001, vs. siNC; #*p* < 0.05, ###*p* < 0.001, ####*p* <

0.0001 vs. siNC+ox-LDL.


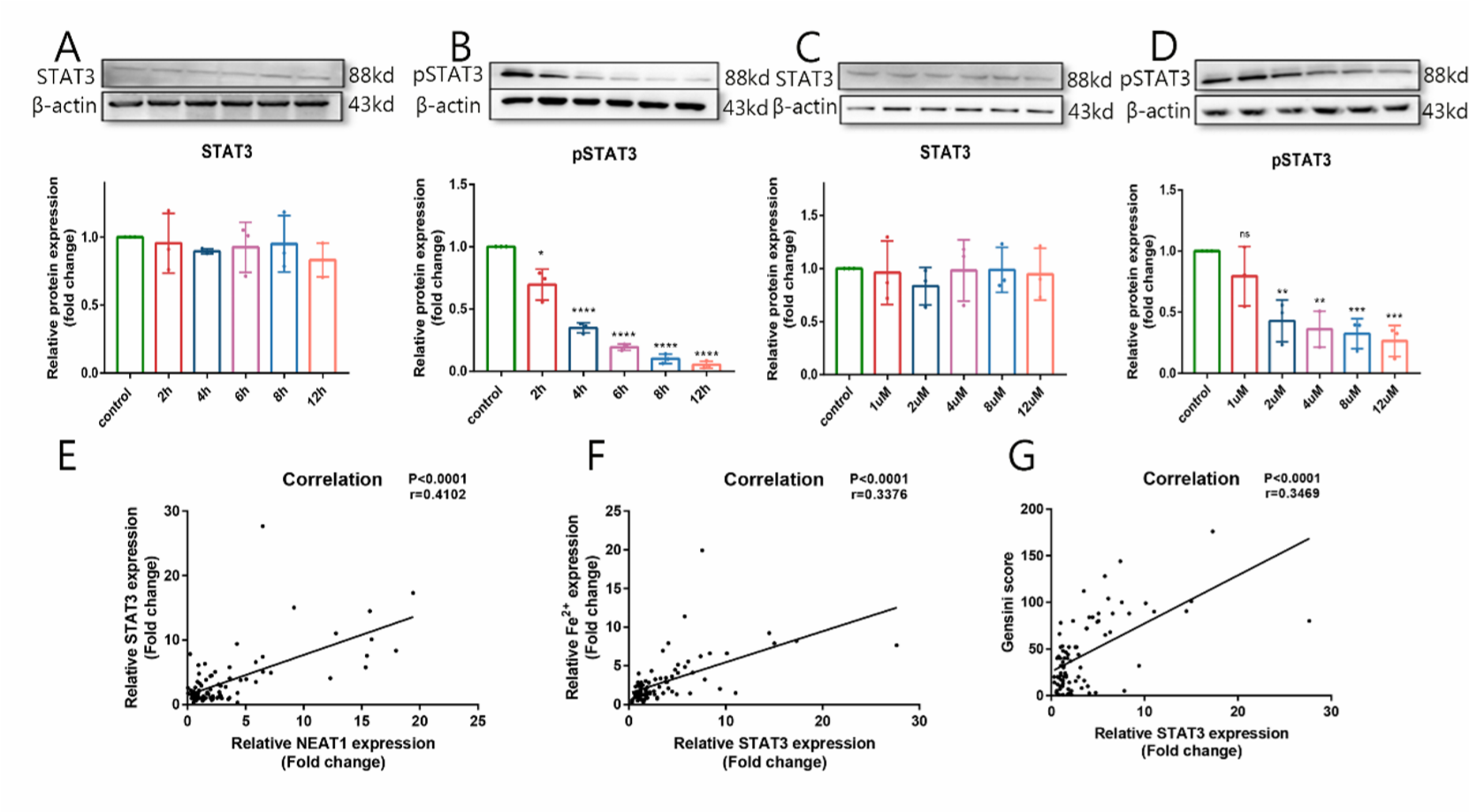


**Figure S3 Optimization of STAT3 inhibitor treatment**

(A, B) THP-1 cells were treated with 1 µM STAT3 inhibitor for 2 h, 4 h, 8 h, 12 h, or 24h. Western blot analysis was conducted to determine STAT3 (A) and pSTAT3 (B) protein expression. (C, D) THP-1 cells were treated with different concentrations of STAT3 inhibitor (1 µM, 2 µM,4 µM, 8 µM, and 20 µM) for 24 h. Western blot analysis was carried out to determine STAT3 (C) and pSTAT3 (D) protein expression. Data are expressed as the mean ± SD. **p* < 0.05, ***p* < 0.01, ****p* < 0.001, *****p* < 0.0001 vs. control. (E–G) PBMCs were isolated from patients with CHD. Spearman correlation analysis was performed (E)NEAT1 and STAT3 expression (F), as well as iron content and STAT3 expression, (G)and between STAT3 expression and Gensini scores. (n = 84).


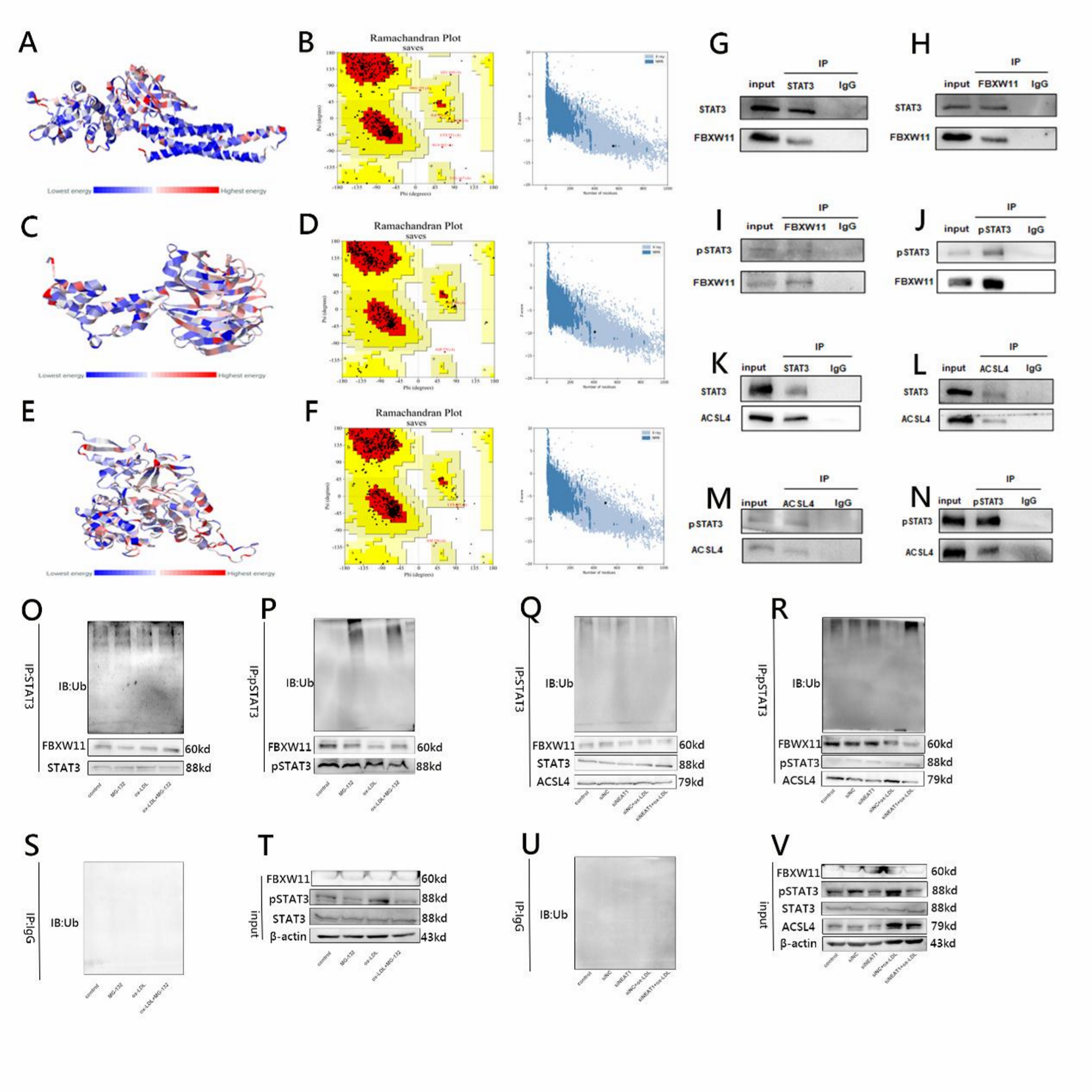
 **Figure S4:** **Co-IP analysis and identification of downstream molecules, and pSTAT3 ubiquitination regulated by NEAT1**

(A) 3D structures of STAT3, (B) PROCHECK server Ramachandran plot analysis and Z-scores of the

3D structures analyzed by ProSA server analysis of STAT3, (C) 3D structures of FBWX11, (D) PROCHECK server Ramachandran plot analysis and Z-scores of the 3D structures analyzed by ProSA server analysis of FBWX11, (E) 3D structures of ACSL4, (B) PROCHECK server Ramachandran plot analysis and Z-scores of the 3D structures analyzed by ProSA server analysis of ACSL4,(G-N) Co-IP analysis of STAT3/FBXW11 (G-H), STAT3(Y705)/FBXW11 (I-J), pSTAT3/ACSL4 (K-L), and STAT3(Y705)/ACSL4,(O-V) IP and drugs was conducted in RAW cells (O)Immunoprecipitation (IP) was conducted to examine ubiquitination of STAT3,(P) and pSTAT3 (Y705) and IgG (S). (T) RAW cells were treated with ox-LDL (100 µg/mL, 24 h) and MG-132 (10 µM, 6 h), alone or in combination. (Q-R) NEAT1 expression was silenced in RAW cells, followed by treatment with ox-LDL (100 µg/mL, 24 h), siNC and siNEAT1, alone or in combination. IP was conducted to examine ubiquitination of STAT3 (Q) and pSTAT3 (Y705) (R) and IgG (U). (V) NEAT1 expression was silenced in RAW cells, followed by treatment with ox-LDL (100 µg/mL, 24 h), siNC and siNEAT1, alone or in combination.


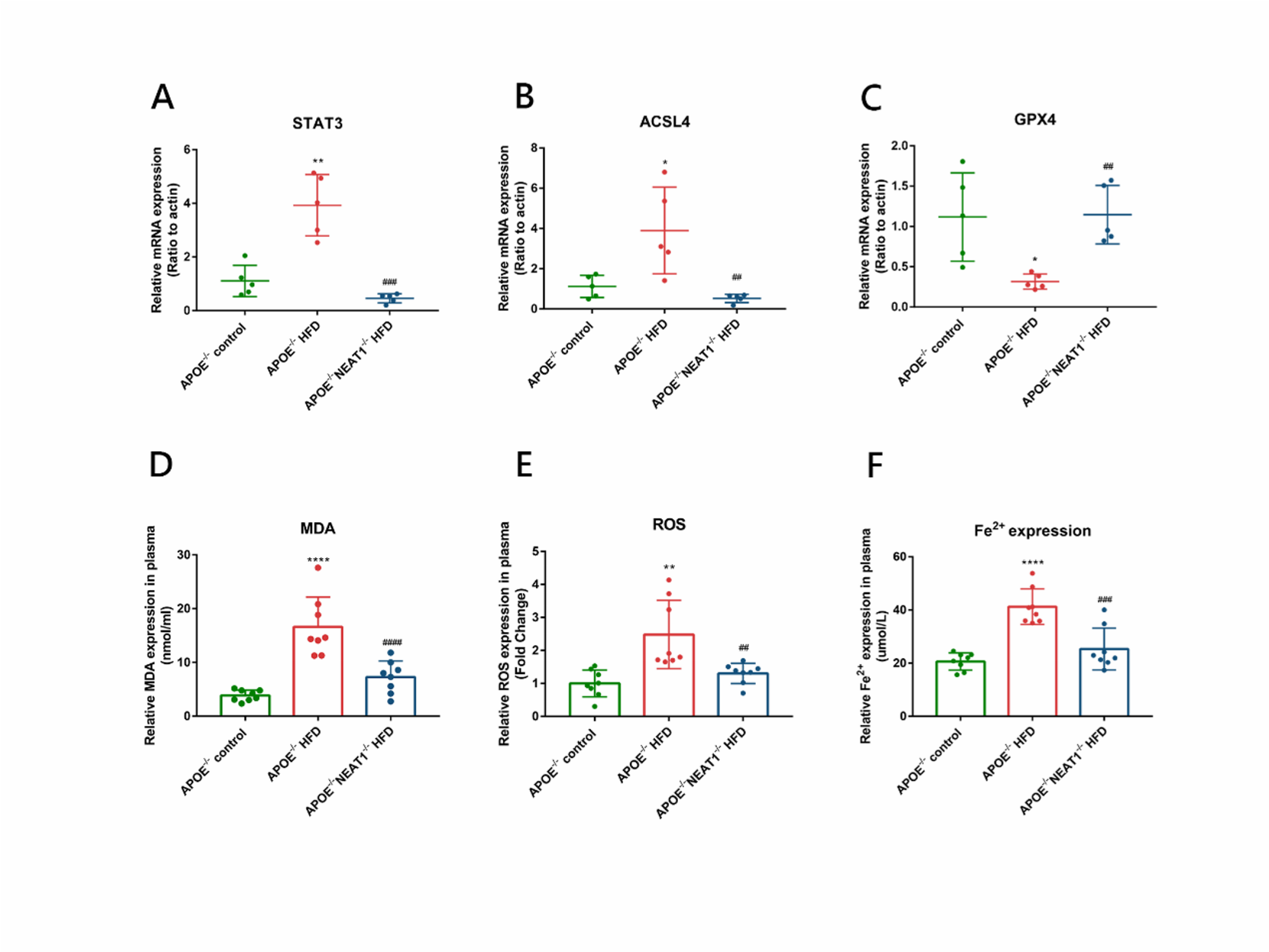


**Figure S5 NEAT1 deficiency prevented HFD-induced ferroptosis and atherosclerosis in APOE^-/-^ mice.**

(A–C) mRNA expression of STAT3, ACSL4, and GPX4 in mouse PBMCs detected by qRT-PCR (n = 5). (D-F) ROS, MDA, and iron levels in the plasma. (n = 8). Data are expressed as the mean ± SD. **p* < 0.05, ***p* < 0.01, *****p* < 0.0001 vs. APOE^-/-^ control; #*p* < 0.05, ##*p* < 0.01, ###*p* < 0.001 vs. APOE^-/-^ control + HFD.


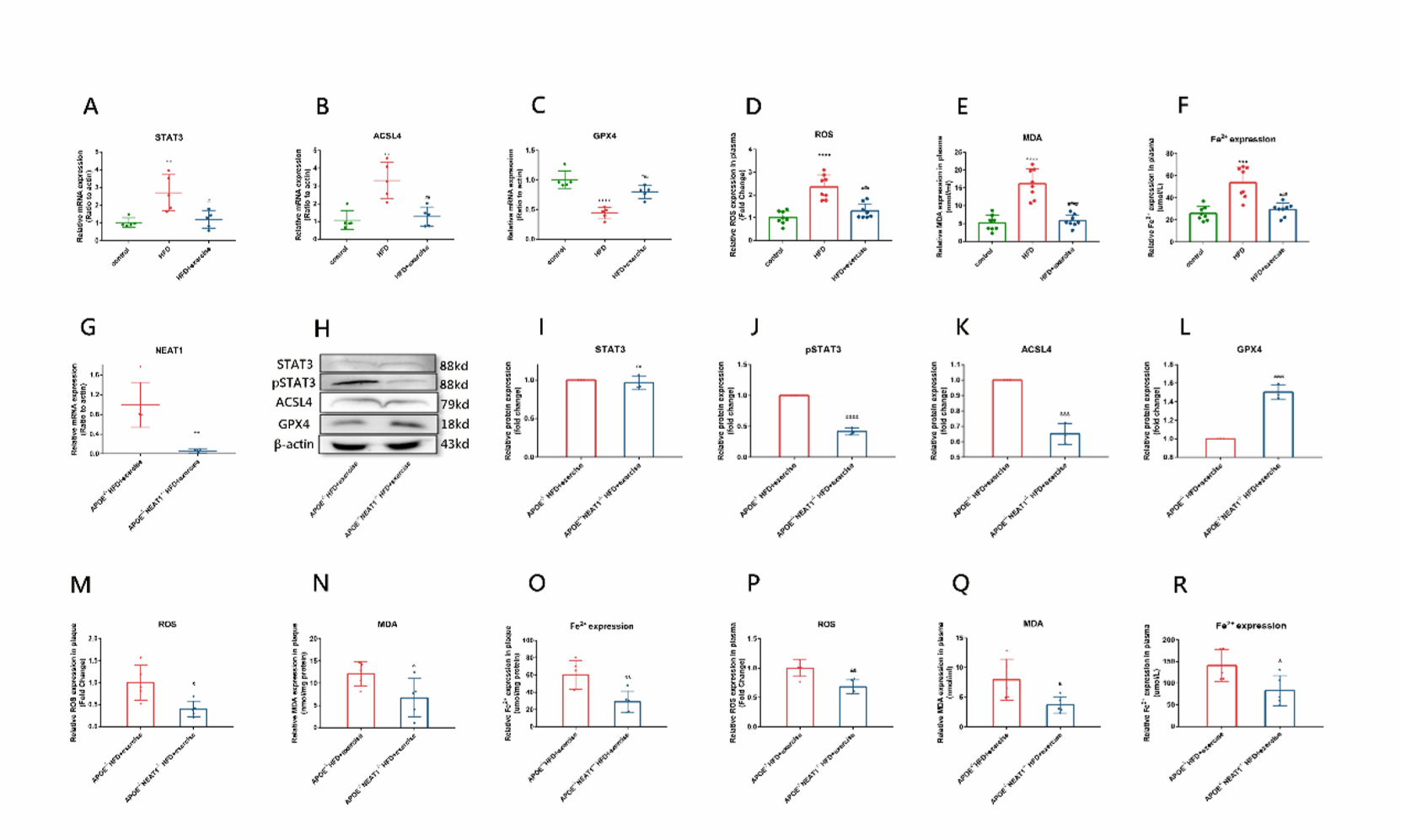


**Figure S6 Exercise reduced NEAT1 expression and prevented HFD-induced ferroptosis and atherosclerosis in mice.**

Eight-week-old APOE^-/-^ and APOE^-/-^NEAT1^-/-^ mice were fed HFD for 16 weeks (n = 8/group). Mice performed aerobic exercise on a treadmill once daily, 5 days per week. (A–C) mRNA expression of STAT3, ACSL4, and GPX4 in mouse PBMCs detected by qRT-PCR (n = 5). (D-F) ROS, MDA, and iron levels in the plasma. (n = 8). (G) NEAT1 expression in PBMCs detected by qRT-PCR. (H) Protein expression of STAT3, pSTAT3, ACSL4, and GPX4 in atherosclerotic plaques detected by Western blot analysis (n =3). (I-L) Quantitative analysis of Protein expression. (M-O) ROS, MDA and iron levels in the atherosclerotic plaques. (n=5) (P-R) ROS, MDA and iron levels in the plasma. Data are expressed as the mean ± SD. **p* < 0.05, ***p* < 0.01, ****p* < 0.001, *****p* < 0.0001 vs. APOE^-/-^ + HFD, #*p* < 0.05, ##*p* < 0.01, ###*p* < 0.001 vs. HFD, & *p* < 0.05, &&*p* < 0.01, &&&*p* < 0.001 vs. APOE^-/-^ HFD+exercise. ns, non-significant.


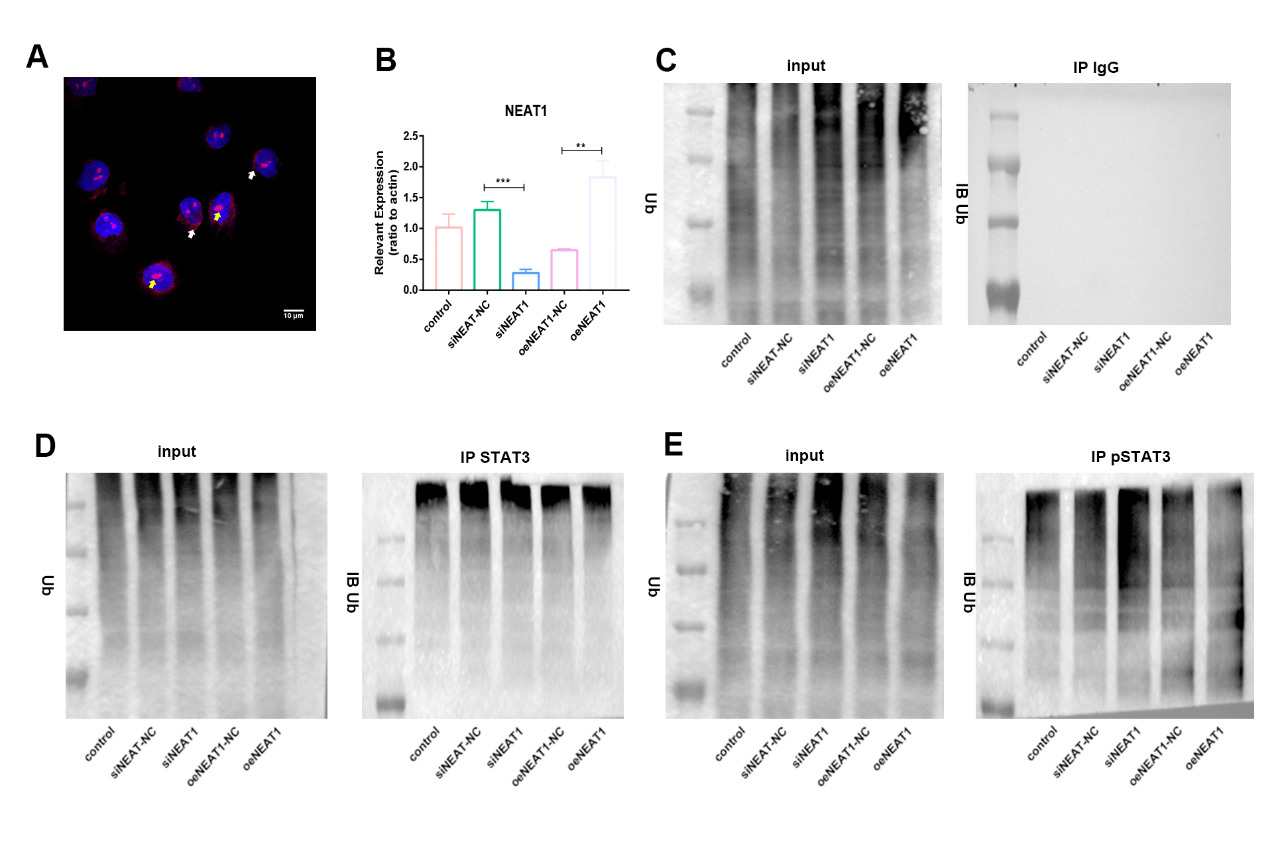


**Figure S7. NEAT1 is predominantly nuclear and selectively regulates pSTAT3 ubiquitination.**

(A) FISH analysis showing nuclear localization of NEAT1 in THP-1 macrophages. Nuclei were counterstained with DAPI. Yellow arrows indicate nuclear localization, and white arrows indicate cytoplasmic localization. Scale bar: 10 μm. (B) qRT-PCR validation of NEAT1 knockdown and overexpression efficiency in THP-1 cells. Data are presented as mean ± SD. ***p* < 0.01, ****p* < 0.001; n = 3. (C–E) Co-IP assays showing that NEAT1 modulation alters the ubiquitination level of phosphorylated STAT3 (pSTAT3) but not total STAT3 in THP-1 cells. MG-132 (10 μM, 6 h) was used to block proteasomal degradation. Input and IP lysates were analyzed by immunoblotting with the indicated antibodies. Representative blots from three independent experiments are shown.

**Table S1**: **Clinical characteristics of human subjects.**

| Characteristics | Control (n = 50) | CHD (n = 84) | CHD + exercise (n = 50) | P-value |
| --- | --- | --- | --- | --- |
| Age (years) | 60 (55.8,64.0) | 64.0 (58.0,69.0) | 64.0 (60.0,67.3) | 0.003 |
| Sex male (n%) | 20 (40.0%) | 40 (47.6%) | 16 (32.0%) | 0.203 |
| TC (mmol/L) | 4.73 ± 0.78 | 4.28 ± 1.01 | 5.18 ± 1.64 | 0.011 |
| TG (mmol/L) | 1.57 ± 1.38 | 1.80 ± 1.58 | 2.30 ± 1.35 | 0.001 |
| HDL (mmol/L) | 1.41± 0.40 | 1.01 ± 0.25 | 2.06 ± 0.99 | < 0.001 |
| LDL (mmol/L) | 2.77 ± 0.68 | 2.66 ± 0.87 | 2.62 ± 0.97 | 0.451 |

Characteristics are described as the median (interquartile interval) and analyzed by the Kruskal–Wallis rank sum test. Patient sex is presented as n (%) and analyzed by the chi-square test. CHD, coronary heart disease; TC, total cholesterol; TG, triglycerides; LDL, low-density lipoprotein cholesterol; HDL, highdensity lipoprotein cholesterol.

**Table S2: PCR primer sequences**

| Genes | Forward | Reverse |
| --- | --- | --- |
| Mus-NEAT1 (genotyping) | CGCAGAGATCCCTCCGCCAC | CACAGTCAGGCCCAGGTGTTCTC |
| Mus-NEAT1-He/Wt | AAAGCAGGAGGCCACTGTGAGC | CAGTCCACCCGTCTCCATCAACTG |
| APOE-WT | GCCTAGCCGAGGGAGAGCCG | TGTGACTTGGGAGCTCTGCAGC |
| APOE-mutant | GCCTAGCCGAGGGAGAGCCG | GCCGCCCCGACTGCATCT |
| Homo-NEAT1 | GGCGAGGTGCCTTTACTACAT | CAACAGCATACCCGAGACTACTT |
| Mus-NETA1 | CTTGTTCTGGGAGCATCAT | CTACACCTTACGCAATCTTCT |
| Homo-ACTB | TCATGAAGTGTGTGACGTGGACATC | CAGCAGGAGCAATGATCTTGATCT |
| Mus-ACTB | CTTCTTTGCAGCTCCTTCGT | CTTCTGACCCATTCCCACC |
| Homo-STAT3 | CTGGCCCCTTGGATTGAGAG | GAAGCGGCTATACTGCTGGT |
| Mus-STAT3 | CAGTTCCTGGCACCTTGGAT | ACGATCCGGGCAATTTCCAT |
| Homo-ACSL4 | TCTGCTTCTGCTGCCCAATT | CGCCTTCTTGCCAGTCTTTT |
| Mus-ACSL4 | CTGGAATGACAGGCCAGTGT | AGGGGCGTCATAGCCTTTCT |
| Homo-GPX4 | ATACGCTGAGTGTGGTTTGC | CTTCATCCACTTCCACAGCG |
| Mus-GPX4 | CCGGCTACAATGTCAGGTTT | ACGCAGCCGTTCTTATCAAT |
| RIPA-NEAT1 | GGCTATGCCTTTACAAGGGAAGTAAG | CCATGGGCTGCACTCAGTAAA |
